# Systematic evaluation of scoring methods for Ki67 as a surrogate for 21-gene recurrence score

**DOI:** 10.1038/s41523-021-00221-z

**Published:** 2021-02-12

**Authors:** Soonmyung Paik, Youngmee Kwon, Moo Hyun Lee, Ji Ye Kim, Da Kyung Lee, Won Jeong Cho, Eun Young Lee, Eun Sook Lee

**Affiliations:** 1 Institute for Personalized Cancer Therapy, Yonsei University College of Medicine, Seoul, South Korea; 2 Department of Pathology, National Cancer Center, Goyang, South Korea; 3 Department of Surgery, Keimyung University Dongsan Hospital, Daegu, Korea; 4 Department of Surgery, Yonsei University College of Medicine, Seoul, South Korea; 5 Department of Surgery, National Cancer Center, Goyang, South Korea

**Keywords:** Breast cancer, Breast cancer

## Abstract

Although Ki67 labeling index is a potential predictive marker for chemotherapy benefit, its clinical utility has been limited by the lack of a standard scoring method resulting in poor interobserver reproducibility. Especially, there is no consensus on the use of average versus hotspot score for reporting. In order to determine the best method for Ki67 scoring and validate manual scoring method proposed by the International Ki67 Working Group (IKWG), we systematically compared average versus hotspot score in 240 cases with a public domain image analysis program QuPath. We used OncotypeDx Recurrence Score (RS) as a benchmark to compare the potential clinical utility of each scoring methods. Both average and hotspot scores showed statistically significant but only modest correlation with OncotypeDx RS. Only hotspot score could meaningfully distinguish RS low-risk versus high-risk patients. However, hotspot score was less reproducible limiting its clinical utility. In summary, our data demonstrate that utility of the Ki67 labeling index is influenced by the choice of scoring method.

## Introduction

OncotypeDx (Genomic Health, Redwood City, CA, USA), a 21-gene breast cancer recurrence score (RS) assay, has both prognostic and predictive value for estrogen receptor positive (ER+)/human epidermal growth factor receptor 2 negative (HER2−)/lymph node negative breast cancer patients^1,2^. Although NSABP B-20 trial demonstrated a statistically significant benefit from adjuvant chemotherapy added to 5 years of tamoxifen in estrogen receptor positive axillary node negative breast cancer, post hoc analyses suggested that the benefit is limited to a subset of patients with high OncotypeDx 21-gene RS^1,2^.

Clinical utility of OncotypeDx was prospectively validated in Trial Assigning Individualized Option for Treatment (TAILORx) trial^3,4^. In TAILORx trial, chemotherapy did not improve distant disease free survival of patients over age 50 with RS below 25 or patients below age 50 with RS below 20^3,4^.

Despite the level I evidence for its clinical utility, high cost has been an impediment to its adoption in developing countries resulting in a significant global health care disparity.

This strongly indicates that further development of a low cost, accessible surrogate for OncotypeDx is necessary.

The meta-analysis of gene expression profiles in breast cancer suggested that the prognostic ability of gene expression-based tests including the OncotypeDx is mainly driven by the expression levels of proliferation genes^5^. Thus, Ki67 labeling index (LI) based on immunohistochemistry has a potential to serve as a surrogate for OncotypeDx.

Several studies have attempted to develop sophisticated algorithms in order to predict the OncotypeDx RS based on pathological features including manually scored Ki67.

For example, Magee Equation Score based on Nottingham grade and on expressions of ER, progesterone receptor (PR), HER2, and Ki67 could be used to identify 71% of patients who need not be tested for OncotypeDX in a prospective validation study of 205 patients^6^.

However, utilization of Ki67 LI has been hampered by poor inter-laboratory reproducibility mainly due to the lack of a standard scoring method as demonstrated by a multicenter reproducibility study conducted by the International Ki67 Working Group (IKWG)^7,8^. Most importantly, there is no agreement as to whether average counts from the entire tumor area or only hotspot counts should be used when hotspots exist within the same tumor. IKWG has since developed and analytically validated a manual scoring method that proved to be reasonably reproducible after rigorous training. In the multicenter analytical validation study with whole sections from 30 cases, only average score met the predefined reproducibility criterion (set at lower 95% confidence interval (CI) > 0.8 for intraclass correlation coefficient (ICC)) with ICC of 0.87 (95% CI = 0.799–0.93)^9^. Hotspot score did not meet the predefined success criterion with ICC of 0.83 (95% CI = 0.74–0.90)^9^. Based on these results, IKWG concluded to move forward with evaluation of clinical utility of average score.

Utilization of image analysis programs achieved the same degree of ICC for average score (0.89, 95% CI = 0.81–0.96) as manual scoring by rigorously trained scorers when applied to core biopsy specimens from 30 patients^10^. However, ICC for hotspot score was only 0.77 (95% CI = 0.63–0.88). On the other hand, automated hotspot identification algorithm deployed by a commercial image analysis platform (Visiopharm HALO) was reproducible and provided superior prognostic information than manual Ki67 counts in single institution studies^11,12^.

In order to determine the best scoring method for Ki67 LI and to evaluate the feasibility of utilizing an open source image analysis software for objective scoring, we utilized QuPath^13^ for unbiased analysis of whole sections stained with Ki67 from a cohort of 240 cases previously tested for OncotypeDx. Similar to the method employed by a commercial image analysis platform described by Thakur et al.^12^, invasive tumor areas were divided into square grids of roughly the size of ×400 magnification microscopic fields and Ki67 LI of tumor cells within each grid were generated. The highest Ki67 LI was then recorded as the hotspot score, while average score was the average of Ki67 LIs from all grids. While manual scoring was not performed, we simulated manual scoring method proposed by the IKWG by selecting total of four grids following the IKWG guideline of random sampling from each staining level. By performing 1000 simulations in silico, we could estimate the median of global average and weighted average scores. We examined the correlation between Ki67 average score and hotspot score, as well as their association with OncotypeDx RS and other clinicopathological features.

## Results

### Systematic scoring of whole section with QuPath public domain image analysis program

In order to avoid subjectivity in selection of the microscopic fields to score and achieve unbiased objective scoring results, we examined the whole Ki67-stained slides from a cohort with available OncotypeDx results from National Cancer Center.

While the grid size of 300 μm was used for primary analysis, we also collected data for grid sizes of 400 and 500 μm. Average 651 (range 7–2920), 423 (range 6–2021), and 290 (range 4–1146) grids were generated per case for each grid size respectively. Annotated data for each grid were exported to a spreadsheet and grids with less than 100 tumor cells were excluded. Mean tumor cell counts were 268.4, 422.8, and 613.9, respectively, for each grid sizes. Ki67 LI was calculated for all grids and maximum Ki67 LI was labeled as the hottest spot score. The average of top five grids were labeled as the hotspot score as was described by Tharkur et al.^12^. Average score was defined as the sum of Ki67-stained tumor cells over the sum of tumor cells from all grids. All three scores were highly reproducible among three grid sizes with correlation coefficient above 0.95. (Supplementary Fig. 1).

### Characteristics of average and hotspot scores

**Fig. 1:**
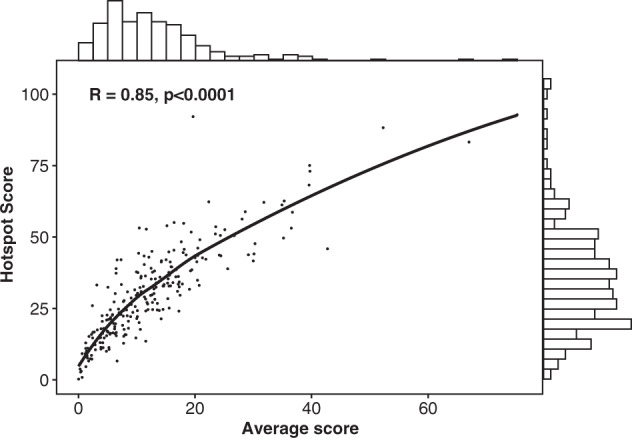
Distribution and correlation between Ki67 labeling indexes. Each dot represents individual cases. Solid line represent Loess smoothing fit of the data. The bar graph on *X-*axis represents distribution of the average score. The bar graph on *Y-*axis represents distribution of the hotspot score. *R* = Pearson correlation coefficient.

Neither of the two Ki67 LIs were normally distributed (Shapiro–Wilk test *p* < 0.0001) but interquartile range was wider for the hotspot score (median of 10.6, 1st quartile 5.5, 3rd quartile 16.1 for the average score; median of 29.1, 1st quartile 17.8, 3rd quartile 29.6 for the hotspot score; median of 32.7, 1st quartile 20.7, 3rd quartile 44.8 for the hottest spot score) (Fig. 1). Average score was heavily right skewed with approximately 50% of cases having score below 10 whereas hotspot or hottest spot scores were less skewed.

### Correlation between average score and hotspot score

We observed a strong correlation between the average and hotspot score (Pearson *r* = 0.85, *p* < 0.0001) with hotspot score about two times the average score (Fig. 1).

### Correlation between Ki67 LI and OncotypeDx RS as continuous variables

In this cohort of 240 ER+ patients, 116 (48.3%), 58 (4.1%), 36 (15.0%), and 30 (12.5%) patients had OncotypeDx RS below 15, 16–20, 21–25, and over 25, respectively (Tables 1 and 2).

**Table 1:** Correlation between Ki67 labeling indexes/tumor grade and 21-gene recurrence score as categorical variables in women age < 50.

Variable	RS category based on TAILORx				Total	*χ*^2^	*P* value
	0–15	16–19	20–25	>25			
Ki67 hotspot							
<10	1	1	3	0	5 (3.3%)	30.08	0.0003
<20	23	14	0	0	37 (24.2%)		
<30	20	8	4	2	34 (22.2%)		
<40	20	9	7	4	40 (26.1%)		
≥40	12	9	6	10	37 (24.2%)		
Ki67 average							
<10	37	19	6	3	65 (42.5%)	47.96	<0.0001
<20	32	17	11	5	65 (42.5%)		
<30	5	5	1	1	12 (7.8%)		
<40	2	0	2	4	8 (5.2%)		
≥40	0	0	0	3	3(1.9%)		
Ki67 hottest spot							
<10	0	1	2	0	3 (1.9%)	27.76	0.0059
<20	14	10	1	0	25 (16.3%)		
<30	22	11	2	1	36 (23.5%)		
<40	19	7	6	4	36 (23.5%)		
≥40	21	12	9	11	53 (34.6%)		
Grade							
1	24	9	2	1	36 (23.5%)	25.58	0.0003
2	46	29	13	7	95 (62.1%)		
3	6	3	5	8	22 (14.4%)		
Grade and Ki67 combined							
Low risk (Grade 1 or Hotspot < 20)	36	18	5	1	60 (39.2%)	11.49	0.0094
High risk (others)	40	23	15	15	93 (60.8%)		
Total	76 (49.6%)	41 (26.8%)	20 (13.9%)	16 (10.5%)	153

**Table 2:** Correlation between Ki67 labeling indexes/tumor grade and 21-gene recurrence score as categorical variables in women age ≥ 50.

Variable	RS category based on TAILORx				Total	*χ*^2^	*P* value
	0–15	16–19	20–25	>25			
Ki67 hotspot							
<10	4	4	3	1	12 (13.8%)	24.71	0.016
<20	12	3	0	0	15 (17.2%)		
<30	14	3	3	2	22 (25.3%)		
<40	6	2	3	5	16 (18.4%)		
≥40	4	5	7	6	22 (25.3%)		
Ki67 average							
<10	27	10	5	2	44 (50.6%)	30.49	0.0023
<20	12	5	6	6	29 (33.3%)		
<30	1	2	4	2	9 (10.3%)		
<40	0	0	1	3	4 (4.6%)		
≥40	0	0	0	1	1(1.1%)		
Ki67 hottest spot							
<10	3	4	2	1	10 (11.5%)	21.41	0.0447
<20	8	3	1	0	12 (13.7%)		
<30	15	2	2	2	21 (24.1%)		
<40	9	3	4	3	19 (21.8%)		
≥40	5	5	7	8	25 (28.7%)		
Grade							
1	9	4	0	2	15 (17.2%)	17.80	0.0067
2	29	13	11	7	60 (69.0%)		
3	2	0	5	5	12 (13.8%)		
Grade and Ki67 combined							
Low risk (grade 1 or hotspot < 20)	23	8	3	2	36 (41.4%)	12.13	0.0069
High risk (others)	17	9	13	12	51 (58.6%)		
Total	40 (46.0%)	17 (19.5%)	16 (18.4%)	14 (16.1%)	87		

Hence according to TAILORx results, 14 (16.1%) of 87 women with age equal or above 50 and 36 (23.5%) of 153 women with age under 50 would be recommended to receive chemotherapy. Additional 41 (26.8%) women age below 50 would be considered for ovarian suppression if considered clinical high risk.

**Fig. 2:**
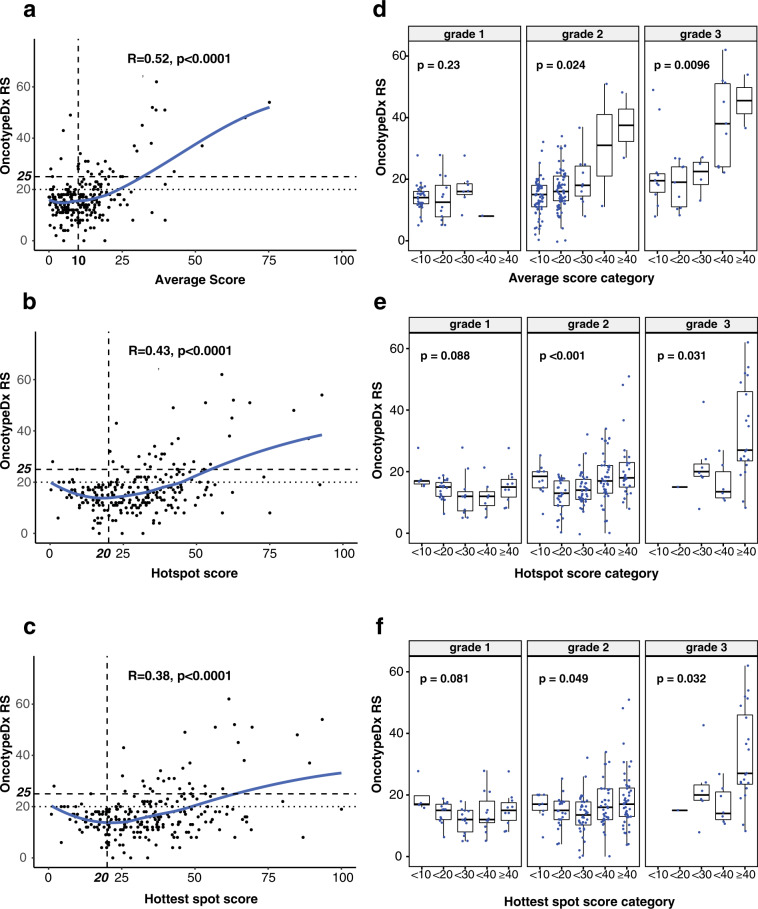
Correlation between 21-gene recurrence score (RS) and Ki67 labeling indexes as continuous and categorical variables. **a** Correlation between Ki67 average score as a continuous variable and OncotypeDx Recurrence Score (RS). Solid line represent loess smoothing fit of the data. Relevant cut points are shown as broken lines with bold legends in each axis. *R* = Pearson correlation coefficient. **b** Correlation between Ki67 hotspot score as a continuous variable and OncotypeDx RS. **c** Correlation between Ki67 hottest spot score as a continuous variable and OncotypeDx RS. **d** Correlation between Ki67 average score as a categorical variable and OncotypeDx RS within each histological grade. Box plot is drawn as follows; each box represents interquartile range with the inside line representing the median value. Whiskers represent maximum (75th percentile + 1.5 × interquartile range) and minimum (25th percentile – 1.5 × interquartile range) values. Each dot represents individual samples. *P* value is from Kruskal–Wallis test. **e** Correlation between Ki67 hotspot score as a categorical variable and OncotypeDx RS within each histological grade. f Correlation between Ki67 hottest spot score as categorical variable and OncotypeDx RS within each histological grade.

Average, hotspot, and hottest spot Ki67 LI showed statistically significant but only modest correlation with RS when examined as continuous variables (Pearson *r* = 0.52, 0.43, and 0.38 respectively, *p* < 0.0001) (Fig. 2a–c). Of note, we observed a poor correlation between Ki67 LIs and RS when average score was below 20 (*r* = 0.17, *p* = 0.127) or when hotspot or hottest spot score was below 40 (*r* = 0.09, *p* = 0.196 and *r* = 0.06, *p* = 0.46, respectively). Therefore, this study confirmed that it is impossible to generate a linear predictive model for RS based on Ki67 Lis alone. The observed non-linearity of Ki67 LIs is consistent with a previous report comparing linearity of immunohistochemistry versus mRNA-based measures of ER^14^.

### Correlation between Ki67 LI and OncotypeDx RS as categorical variables

Correlation between each Ki67 LIs and RS are shown in Fig. 2d–f. As mentioned above, distribution of average scores are right skewed with most of the cases scoring below 20%, whereas hotspot and hottest spot scores are more widely distributed. The data also suggest that it will be difficult to identify a categorical cut point for average score to identify low risk patients.

For 42 (27.5%) tumors with hotspot score below 20 and age < 50, 3 (7.1%) tumors had RS > 20 (Table 1). Thus 3 (8.3%) of 36 patients would miss benefit from chemotherapy if OncotypeDx were regarded as a gold standard.

Among 27 (31.0%) patients with hotspot score below 20 and age ≥ 50, only 1 (3.7%) had RS > 25 (Table 2). Therefore among 14 patients whose age were ≥ 50 and RS > 25, 1 (7.1%) had hostspot score <20 and miss potential benefit from chemotherapy if Ki67 hotspot LI is used for chemotherapy decision instead of OncotypeDx. These data suggest that patients with ER+ tumors with hotspot score below 20 (45% in this cohort), especially if age ≥ 50, may safely avoid chemotherapy. However, if OncotypeDx is used instead, 79% of patients in this cohort would avoid chemotherapy.

The hottest spot score assigned a smaller number of patients to below 20% category compared to the hotspot score (Table 1). For 28 (18.3%) tumors with hottest spot score below 20 and age < 50, 3 (10.7%) tumors had RS > 20. Among 22 (25.2%) patients with hottest spot score below 20 and age ≥ 50, only 1 (4.5%) had RS > 25 (Table 2). While cut-off 30% can be considered without greatly increasing false positivity (classifying high RS tumors as low risk), the proportion of cases that miss potentially beneficial treatment increases from 7 to 21% for age ≥ 50 and 8 to 17% for age <50, thus would not be clinically meaningful.

For average score, among 65 (42.5%) tumors with score below 10 and age < 50, 9 (13.8%) had RS > 20 (Table 1). On the other hand, among 44 (50.6%) tumors with score below 10 and age ≥ 50, 7 (15.9%) had RS > 25 (Table 2). Accordingly, 9/36 (25%) and 2/14 (14.3%) of patients respectively for age group below and above 50 would miss potential benefit from chemotherapy if cut off of 10 were to be used for average score.

In sum, our data highlight the contrasting results among different scoring methods and only the hotspot score may have a reasonable clinical utility for prescreening before OncotypeDx testing.

### Tumor grade, progesterone receptor, and RS

In this study cohort, 51 (21.2%), 155 (64.6%), and 34 (14.2%) tumors were classified as grade 1, 2, and 3, respectively.

Higher tumor grade was associated with higher RS (*p* < 0.001 ANOVA). Intriguingly, the correlation between Ki67 LI and RS increased according to tumor grade (Pearson correlation coefficient −0.047, 0.446, and 0.575 for grade 1, 2, and 3, respectively, for average score). For grade 1 tumors in patients with age under 50 (*N* = 36), three tumors had RS above 20 (Table 1). For grade 1 tumors in age ≥ 50 (*N* = 15), two tumors had RS > 25 (Table 2). Thus, using grade 1 as a prescreening, 5/51 (9.8%) will be missing potentially beneficial chemotherapy.

Unlike other studies showing inverse correlation between progesterone receptor and OncotypeDx RS^6^, no statistically significant association was found between estrogen or progesterone receptor levels in our cohort (Supplementary Figs. 4 and 5). This might be due to the inherent bias in the cohort selection, since OncotypeDx is not covered by the national health insurance in Korea.

### Combination of Ki67 LI and tumor grade

Based on the above results, we classified tumors with grade 1 or Ki67 hotspot score below 20 as low risk and then examined association with RS risk categories; thus, 96 (40.0%) cases were categorized as low risk.

**Fig. 3:**
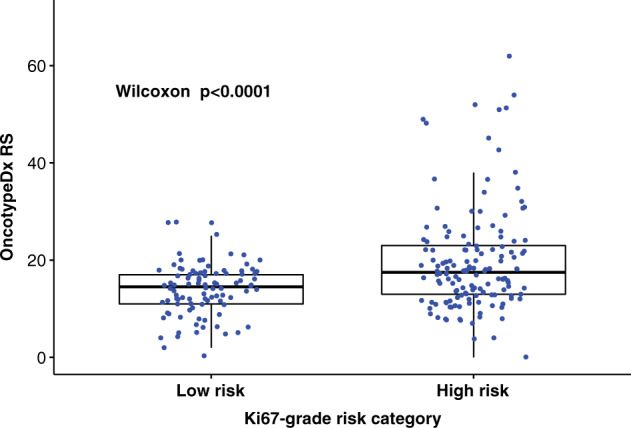
OncotypeDx Recurrence Score (RS) according to Ki67-grade combined risk. Categories (low risk = grade 1 or Ki67 hotspot <20, high risk = rest). Box plot is drawn as follows: each box represents interquartile range with the inside line representing median value. Whiskers represent maximum (75th percentile + 1.5 × interquartile range) and minimum (25th percentile–1.5 × interquartile range) values. Each dot represents individual samples.

Among them 11 (11.5%) tumors had RS above 20, and 3 (3.1%) had RS above 25. When broken down to age groups, among 153 women with age < 50, 60 (39.2%) were classified as low risk and among them 6 (10.0%) had RS > 20. While misclassification rate is 10%, since 36 of 156 patients had RS > 20 and would be offered chemotherapy if OncotypeDx were used for decision making, 6/36 (16.6%) would miss potential benefit from chemotherapy if Ki67 and grade were used for decision (Table 1). For 87 women with age ≥ 50, 36 (41.4%) were classified as low risk and among them 2 (5.6%) had RS > 25. Since 14 of 87 had RS > 25, 2/14 (14.3%) would miss potential benefit from chemotherapy (Table 2). On the whole, by using Ki67 LI and tumor grade, 8 (8.3%) of 96 patients would be falsely classified as low risk if OncotypeDx is regarded as a gold standard (Fig. 3).

### Impact of grid size on Ki67 scores

Our primary analysis was conducted with ki67 scores obtained with QuPath image analysis with grid size 300 × 300 μm. Using a commercial image analysis system, Thakur et al.^12^ used grid size of 500 × 500 μm. We examined if different grid size (hence, number of tumor cells examined) influence the results. For all cases, we repeated analysis with grid sizes of 400 × 400 and 500 × 500 μm. Average scores were nearly identical with Pearson correlation coefficient ranging 0.999–1.000, while there was minor variability for hotspot or hottest spot scores with Pearson correlation coefficient 0.980–0.989 for hotspot score and 0.951–0.965 for hottest spot score (Supplementary Fig. 1).

### Reproducibility of QuPath image analysis based ki67 scores

QuPath image analysis first identify positive and negative stained cells and then use machine learning based cell classification into various cellular components. While this process is robust, in some cases cellular classification may be less than ideal especially when there are proliferating immune cells. Therefore, one would expect some degree of interobserver and intraobserver variability in scoring result unlike results reported in the literature for image analysis systems.

We originally performed the image analysis with grid size for each case selected subjectively based on the tumor size, tumor cellularity, and staining heterogeneity. Average and hottest spot score was collected. Later we reanalyzed all cases with formal method as described in this report with fixed grid size. Since there was a 1 year gap between two readings, we could use the original scoring data as sort of a real world example and compare to the formal data that we generated in a rigid fashion to assess the variability.

As reported by others, intraobserver correlation was excellent with coefficient of 0.9176 (95% CI 0.8950–0.9355) for average score, while correlation was not as good for the hottest spot score with coefficient of 0.8104 (95% CI 0.7620–0.8498). Correlation between original hottest score and formal hotspot score was better with coefficient of 0.8575 (0.8199–0.8877) (Supplementary Fig. 1).

### Simulation of IKWG scoring method

We simulated IKWG global average score and weighted average score^9^ taking advantage of comprehensive grid-based image analysis data collected from this study as detailed in the “Methods” section. We repeated simulation 1000 times and recorded data ranges, median scores, and confidence intervals. Median of weighted average score showed excellent correlation with average score by image analysis (Pearson’s *r* = 0.998) (Supplementary Fig. 2a, b). The global average score was less robust (Pearson’s *r* = 0.941) (Supplementary Fig. 2c, d). Thus our data suggests that weighted average score is a better method. However clinical utility of IKWG average scores may be questionable since it is right skewed and cut off for identifying OncotypeDx RS low-risk tumors is difficult to establish (Supplementary Fig. 3).

## Discussion

Although OncotypeDx is the only gene expression-based test with prospectively] validated clinical utility for prediction of chemotherapy benefit (level I evidence), many other validated prognostic tests exist, including Mammaprint, Prosigna, Breast Cancer Index, Endopredict, and Genomic Grade Index. These tests are performed with diverse technical platforms and with little overlap of constituent genes among them. A meta-analysis of microarray gene expression profiling data by Wirapati et al.^5^ suggested that all published prognostic algorithms including OncotypeDx identify low proliferating ER+ tumors as low risk group. Therefore, despite lack of level I evidence through marker based prospective randomized trials, in 2016 American Society of Clinical Oncology has endorsed the clinical utility of other gene expression based prognostic algorithms in selecting patients who do not need chemotherapy.

On the other hand, it should be noted that unlike in meta-analyses setting in which all cases were normalized together and expression levels of each genes were standardized, in real-world individual patient level testing, expression levels of component genes vary among different tests since they use different technical platforms (such as QRT-PCR versus nCounter assay) and genes are normalized using different set of internal reference genes. Hence, for an example, ESR1 mRNA measurement by OncotypeDx and Prosigna from the same tumor may not correlate very well. Indeed, in a prospective comparison study of 313 patients (OPTIMA Prelim Trial), agreement among OncotypeDx, Mammaprint, and Prosigna was modest with Kappa statistics ranging from 0.28 (between Prosigna and OncotypeDx) to 0.50 (between Mammaprint and OncotypeDx)^15^. For example, if OncotypeDx were to be regarded as gold standard, among 50 tumors with RS over 25, 6 (12%) were misclassified as low risk by Mammaprint, and 8 of 52 with RS over 25 (15.4%) were misclassified as low risk by Prosigna. Conversely among 247 patients with RS below 26 (who would be spared from chemotherapy if age above 50), 70 (28.3%) and 59 (23.9%) were misclassified as high risk by Mammaprint and Prosigna, respectively.

Therefore, in current clinical practice setting where all these tests are now endorsed by ASCO for selection of patients for no chemotherapy, if a test has above 75% concordance with OncotypeDx risk categories and if patients classified as low risk have 10-year distant disease free survival above 90%, the test may have a clinical utility equal to gene expression based tests. In the Optima Prelim Trial, OncotypeDx and other gene expression-based tests were compared to IHC4, a clinicopathological prognostic algorithm which incorporates Ki67 LI. IHC4 misclassified 11/50 (22%) with RS over 25 as low/intermediate risk and 33 of 207 (15.9%) with RS below 26 as high risk. The Kappa statistic for agreement with OncotypeDx was 0.53, which was not worse than Mammaprint or Prosigna.

Although Ki67 LI provides a simple and low-cost measure of proliferation index, its clinical utility has been hampered by the lack of a standard scoring method. For breast cancer, cut off of 14 or 20 has been employed in most published studies. However, detailed method for scoring has been lacking in most of them. According to an international round robin study conducted by the IKWG, scoring method was the main source of variability among laboratories^7^. Since then, IKWG have conducted a series of studies to demonstrate that after a rigorous training for a standardized scoring method, reasonable intraclass concordance rate could be achieved for average score for whole section^9^. However, ICC for hotspot score did not meet the predefined acceptance criteria. While promising, there were only 30 cases examined in the latter study. IKWG also evaluated 10 different image analysis systems including QuPath and found them to achieve the same degree of ICC as in manual scoring by the pretrained pathologists^10^. Thakur et al used a commercial image analysis system to systematically analyze the whole section of 300 cases by dividing tumor area into 500-μm-sized square grids and score each to derive global average and hotspot scores^12^. They reported nearly perfect ICC between two pathologists (Pearson’s *r* = 0.984). Random forest model built with Ki67 and other clinic-pathological features identified OncotypeDx high and low risk patients with high accuracy. However, since the latter study excluded intermediate-risk group from evaluation and model was not validated in an independent cohort, real world performance is not clear.

Using a nearly identical approach to Tharkur et al., but employing a public domain image analysis program QuPath, we systematically compared three scoring methods for Ki67 LI, i.e. average, hotspot, and hottest spot counts. Through grid overlay generation and counting within each grids, we were able to process the whole section image for both average and hotspot counts without bias. Machine learning feature of QuPath allowed scoring of only tumor cells. Interestingly, hotspot counts were roughly twice of average counts. These data may partly explain the diverse cut points reported in the previous studies and underscore the need to detail scoring methods when reporting Ki67 LI. Since hotspot score has a wider interquartile range, hotspot score may be a better method than average score. However, reproducibility needs to be improved for hotspot scoring—which can be achieved by development of an automatic hotspot algorithm for image analysis. The current approach of hotspot identification relies on selecting the highest scoring grid which is influenced by the size of the grid (Pearson *r* ranges from 0.95 to 0.98; Supplementary Fig. 1) As continuous variables, both average and hotspot Ki67 LI showed statistically significant but only modest correlation with RS (Pearson *r* = 0.52 and 0.43 respectively, *p* < 0.0001), making it impossible to reliably predict RS with Ki67 LI alone. However, in a pragmatic approach, combination of Ki67 hotspot score as categorical variable and tumor grade may identify approximately 40% of patients who do not need OncotypeDx and safely skip chemotherapy—since only about 8% of them would miss out potential benefits from chemotherapy.

We performed 1000 times simulation of IKWG scoring method by way of randomly picking scored grids from each staining level according to IKWG guideline. The result shows that when done properly (represented by median score from simulation) the IKWG weighted average scoring method closely approximate global average score from QuPath image analysis (Pearson’s *r* = 0.998). Correlation between IKWG global average score and image analysis average score was lower with Pearson correlation coefficient of 0.940. Since IKWG analytical validation study was conducted with only 30 cases with rigorous guideline and training, its performance in real world is yet to be demonstrated. More importantly, due to the fact that IKWG score is an average score, its clinical utility as a predictive marker for chemotherapy benefit needs further validation deducing from our result.

In summary, our data demonstrate that the clinical utility of Ki67 labeling index may be influenced by the choice of scoring method. Therefore, reporting of the Ki67 results should include a detailed description of how the scoring was performed. Although hotspot scores may have a better clinical utility, its reproducibility needs to be improved.

## Methods

### Subjects

A total of 240 subjects with ER-positive breast cancer were analyzed in the study. All patients diagnosed of ER+ breast cancer at Korean National Cancer Center and tested for OncotypeDx between 2011 and 2015 were included in the study.

### Ethics approval and consent to participate

This study was approved by the institutional review board of Yonsei University College of Medicine (1-2014-0067). All participants signed informed consent to allow use of routine surgical pathology specimens for research. Signed consent was obtained from the patient for a free availability of the published photomicrographs in this manuscript on the internet.

### Immunohistochemistry

As part of a routine immunohistochemistry panel, ER (cline SP1, Ready to Use, Ventana Medical Systems, Inc., Tucson, AZ, USA), PR (clone 1E2, Ready to Use, Ventana), and Ki67 staining (1:200, clone MIB-1, DAKO, Glostrup, Denmark) was performed at National Cancer Center using the Ventana platform.

### Digital image analysis

Slides were scanned with Aperio ScanScope with ×20 objective lens (Leica Biosystems, Buffalo Grove, IL, USA).

**Fig. 4:**
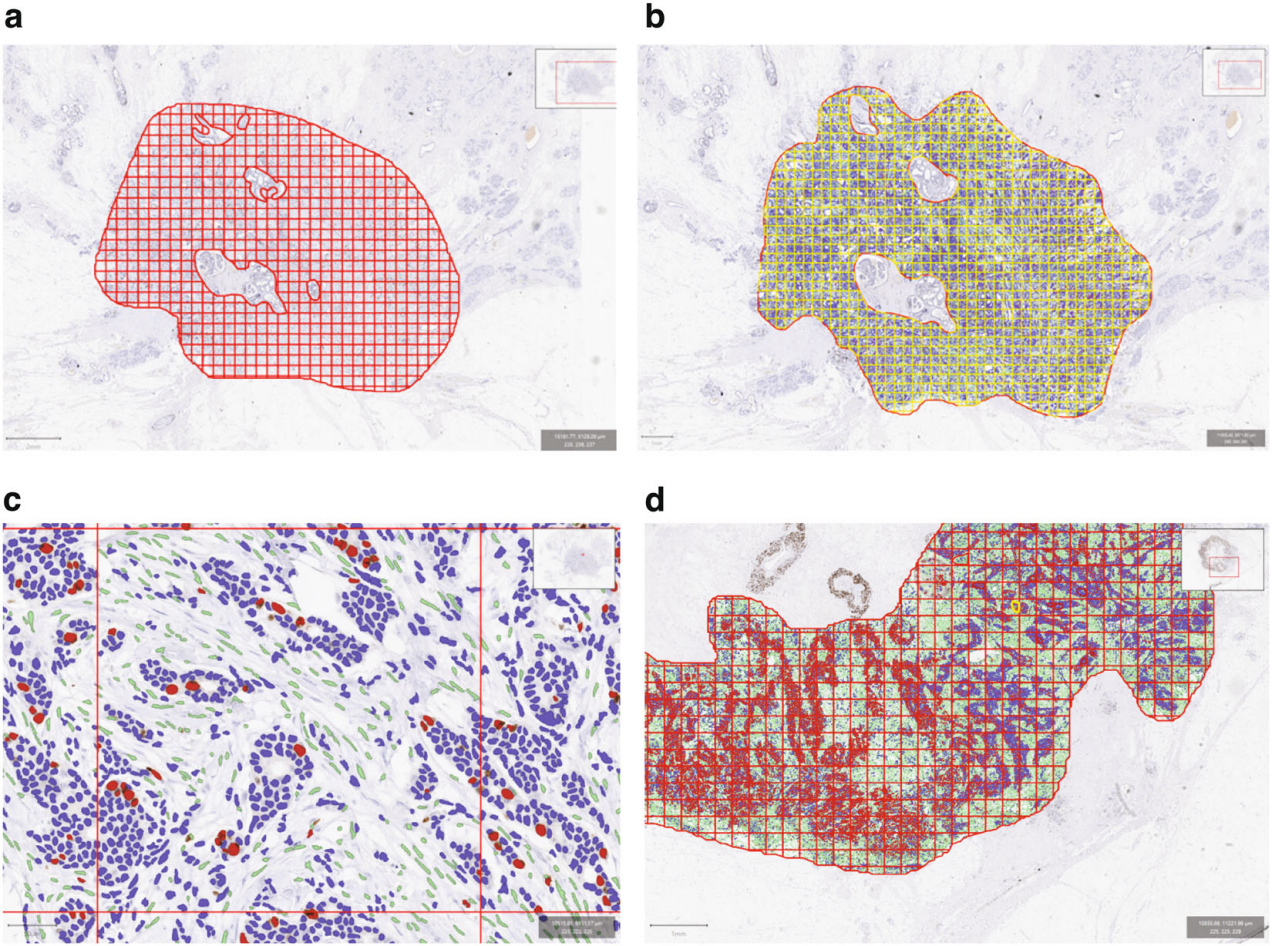
Schema of QuPath image analysis steps. **a** After entire invasive tumor area is identified as a region of interest using a hand drawing tool, 300 × 300 μm grids were generated to fill the region of interest. **b** After positive cell detection within each grid, cell classifier is generated based on annotation of representative tumor and host cells, and applied to all grids. **c** Magnified view of a single grid after cell classifier is applied (right); negative tumor cell nuclei (blue), positive tumor nuclei (red), stromal cell nuclei (green). **d** An example of within-tumor heterogeneity of ki67 staining with left side showing nearly 100% cells staining while right side show lesser staining.

QuPath (version 0.2.0.), an open source image analysis software, was used^13^. Similar to a commercial image analysis platform described by Thakur et al.^12^, square grids were created to fill the entire invasive tumor areas drawn by a pathologist (Fig. 4a). We used grid sizes of 300, 400, and 500 μm. Average 651 (range 7–2920), 423 (range 6–2021), and 290 (range 4–1146) grids were generated per case for each grid size, respectively. Grids containing ductal carcinoma in situ or lymphoid aggregates, as well as those containing staining artifacts were manually excluded upon visual inspection of the grids.

Following the positive cell detection with single threshold to identify Ki67-stained cells, case specific random forest cell classifier was created by identifying representative tumor cells and stromal cells, and applied to all grids (Fig. 4b, c). Annotated data for each grid were exported to a spreadsheet and grids with less than 100 tumor cells were excluded. Ki67 LI was calculated for all grids and maximum Ki67 LI was labeled as the hottest spot score. Average of top five grids were labeled as the hotspot score as was described by Tharkur et al.^12^. Average score was defined as the sum of Ki67-stained tumor cells over the sum of tumor cells from all grids.

A step by step instruction with images is detailed in the supplementary file.

In brief, after importing the scanned image into QuPath, following steps were taken (Supplementary Note 1 for detailed explanation) (Fig. 4):

1. Draw a region of interest (ROI) manually over entire tumor area.
2. Create a grid with size 300 × 300 μm (Analyze → Region identification → Tiles and super pixels → Create tiles → Tile size 300 μm, Trim to ROI: true, Make annotation tiles: true, Remove parent annotation: false → Run) (Fig. 4a).
3. Set positive cell detection with single threshold (Analyze → Cell analysis → Positive cell detection → adjust parameters based on Optical Density Sum, Score compartment: Nucleus DAB OD Mean with single threshold (Fig. 4b).
  *Although default parameters for nuclei detection worked without modification for most cases, for cases with faint Hematoxylin staining or open chromatin pattern (nuclear grade 3), nuclear detection parameters were adjusted to best match the nuclear outline of tumor cells.
4. Create and apply random forest cell classifier after manually annotating representative tumor and non-tumor cells (Fig. 4b).
5. Export annotated data to a spreadsheet.
6. Exclude of grids with less than 100 tumor cells.
7. Calculate average score (the highest score is the hottest spot score, hotspot score is the average of top five scores).
8. steps 2 to 6 were repeated with grid sizes of 400 × 400 and 500 × 500 μm.

### Simulation of IKWG global average and weighted average scores

While manual scoring was not performed, we simulated manual scoring method proposed by the IKWG as follows using a code written in R programming language:

1. Calculate Ki67 score range for each case by subtracting minimum from maximum score.
  Dividing the range by 3 to determine the cut offs for low, medium, and high scores for each case.
2. Assign each grids to the four scoring levels (zero, low, medium, high) and estimate the proportion of each scoring levels by dividing the number of grids assigned to each level by the number of all grids.
3. Randomly select grid to score from each scoring level according to the rule provided by the IKWG, resulting in a total of fout girds to score.
4. Global average score is calculated as total number of positive cells divided by total number of tumor cells with the four selected grids.
5. Weighted average score is calculated by summing the scores of grids from each scoring levels multiplied by the proportion of respective scoring levels.

### Reporting summary

Further information on experimental design is available in the Nature Research Reporting Summary linked to this paper.

## Supplementary information

Supplementary material

Reporting Summary Checklist

## Data Availability

The Ki67 image analysis data supporting the findings of this study are publicly available in the figshare repository: 10.6084/m9.figshare.13378478^16^. The scanned microscopic images can be obtained from the corresponding author upon reasonable request. For data access requests, please contact S.P., Yonsei University College of Medicine, email address: soonmyungpaik@yuhs.ac.

## Code availability

Custom R code is provided in Supplementary Note 2.
