# Supplementary material for: Systematic evaluation of scoring methods for Ki67 as a surrogate for 21-gene recurrence score
Source: NPJ Breast Cancer. 2021 Feb 12;7:13. doi: 10.1038/s41523-021-00221-z (PMC7881194; doi:10.1038/s41523-021-00221-z)
Supplement: Supplementary file 1 — Supplementary material [file 41523_2021_221_MOESM1_ESM.pdf]

Supplementary Information for Paik et al.

Supplementary Note 1. Detailed method for QuPath image analysis …………………………………..2

Supplementary Note 2. R code for data analysis ………………………………………………………….12

Supplementary figure 1. Reproducibility of image analysis ………………………………………………..16

Supplementary figure 2. Simulation of International Ki67 Working Group scoring method..20

Supplementary figure 3. Correlation between the International Ki67 Working Group average scores and OncotypeDx recurrence score……………………………………………………………………..21

Supplementary figure 4. Correlation between estrogen receptor levels and OncotypeDx RS ……..23

Supplementary figure 5. Correlation between progesterone receptor levels and OncotypeDx RS ………..23

Supplementary Note 1. – Detailed method for QuPath image analysis

1) Import whole slide image file to QuPath

2) Draw a region of interest (ROI) manually over the entire tumor area and mouse click at a point outside the ROI (color of the ROI will change).

Note: Do not move the window when ROI is indicated with yellow border – this will result in moving of the ROI to different area. You can click outside the ROI so that ROI is now highlighted with red line, then safely move around.

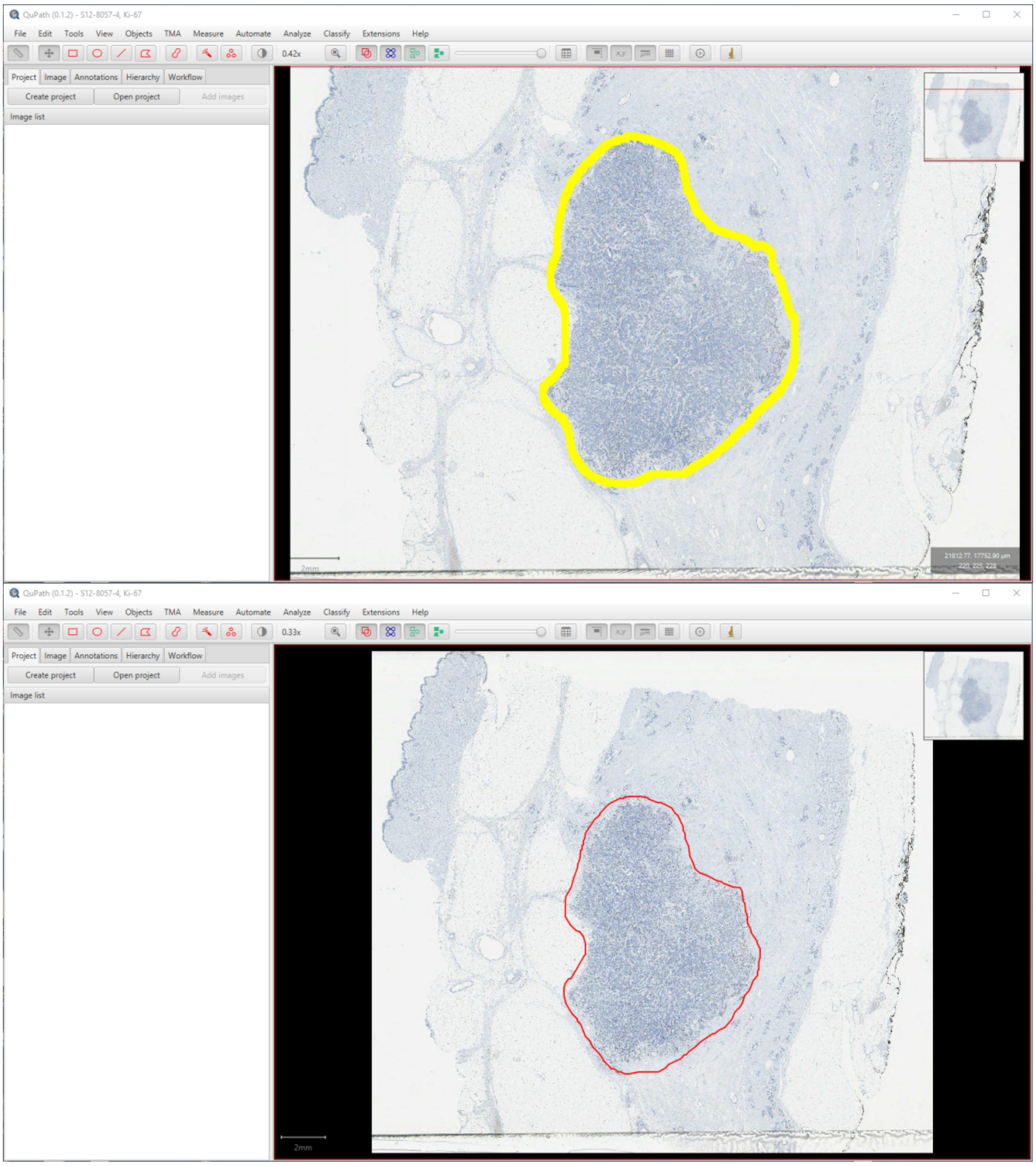

3) From the tool bar, select Analyze → Preprocessing → Estimate stain vectors → select Yes on pop up window → select OK on Visual Stain Editor pop up screen.

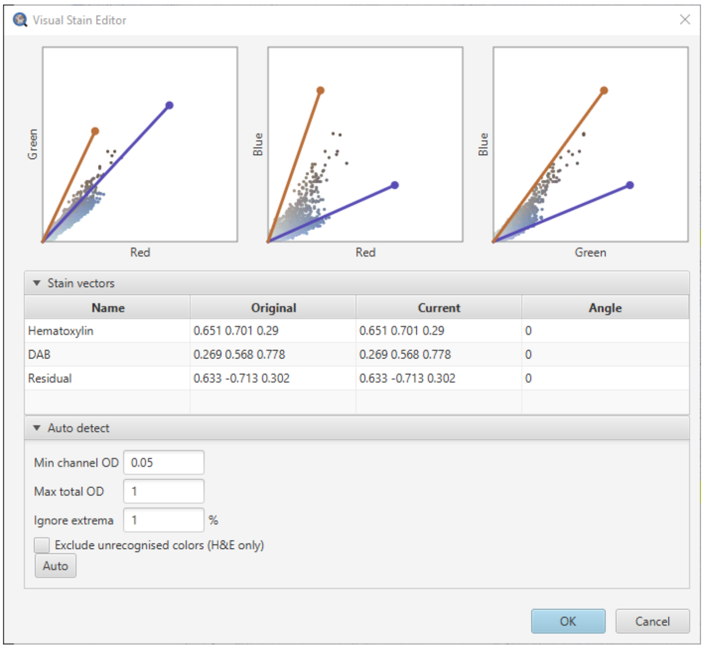

4) Create a grid with size 300x300 or 400x400 micrometer (From tool bar select Analyze → Region identification → Tiles and super pixels → Create tiles → Tile size 300 micrometer, Trim to ROI: true, Make annotation tiles: true, Remove parent annotation: false → Run)

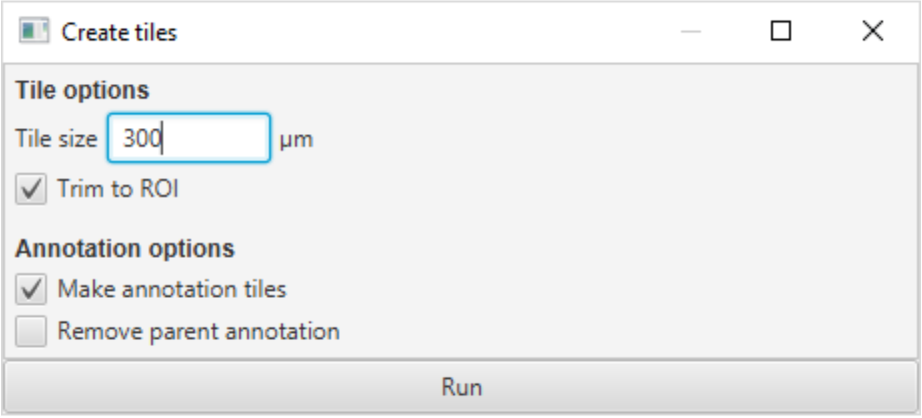

5) Select Annotations on Process Regions pop up window, and click OK

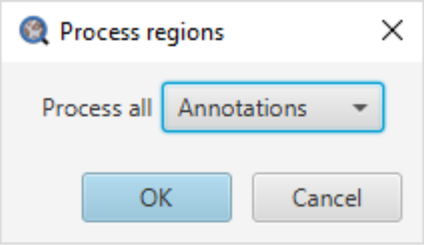

6) The window will now show the following: The ROI will show red colored grids. And the annotation tab of the left menu bar will show polygon with number of objects (which is number of grids created) followed by tiles with serial numbers

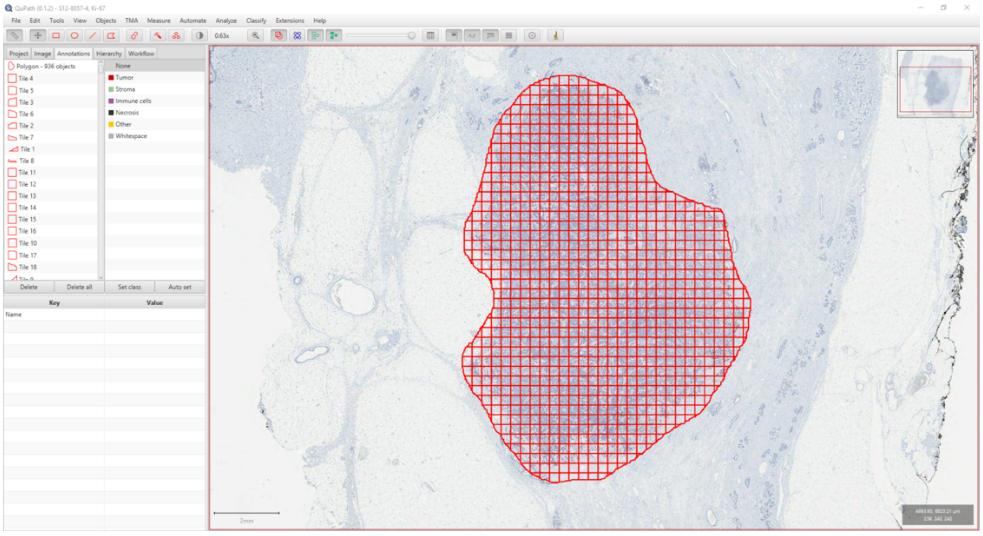

Here is a zoomed view of the annotation tab.

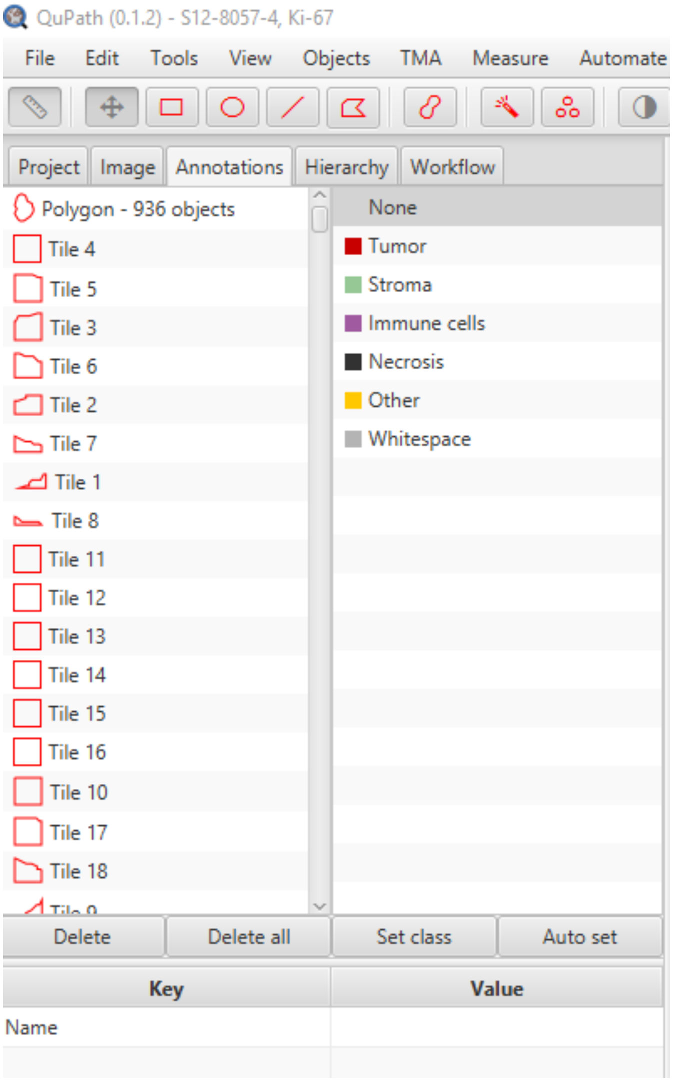

7) Select only tiles from the annotation tab by shift-right mouse click over the tiles

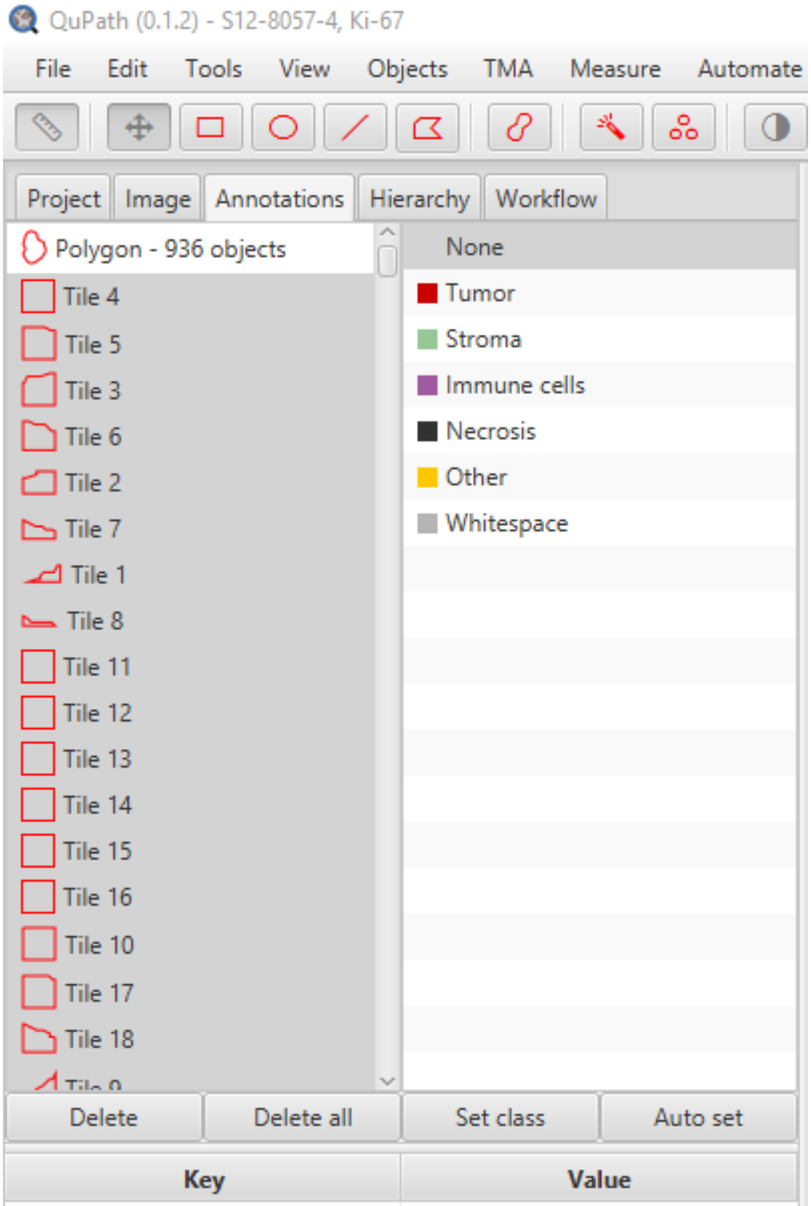

8) perform Positive cell detection with single threshold (From the tool bar, select Analyze → Cell analysis → Positive cell detection → Run → adjust parameters based on hematoxyin staining intensity, Score compartment: Nucleus DAB OD Mean with single threshold → Run → Select Annotations on Process Regions pop up window → click OK.. This will take a while.

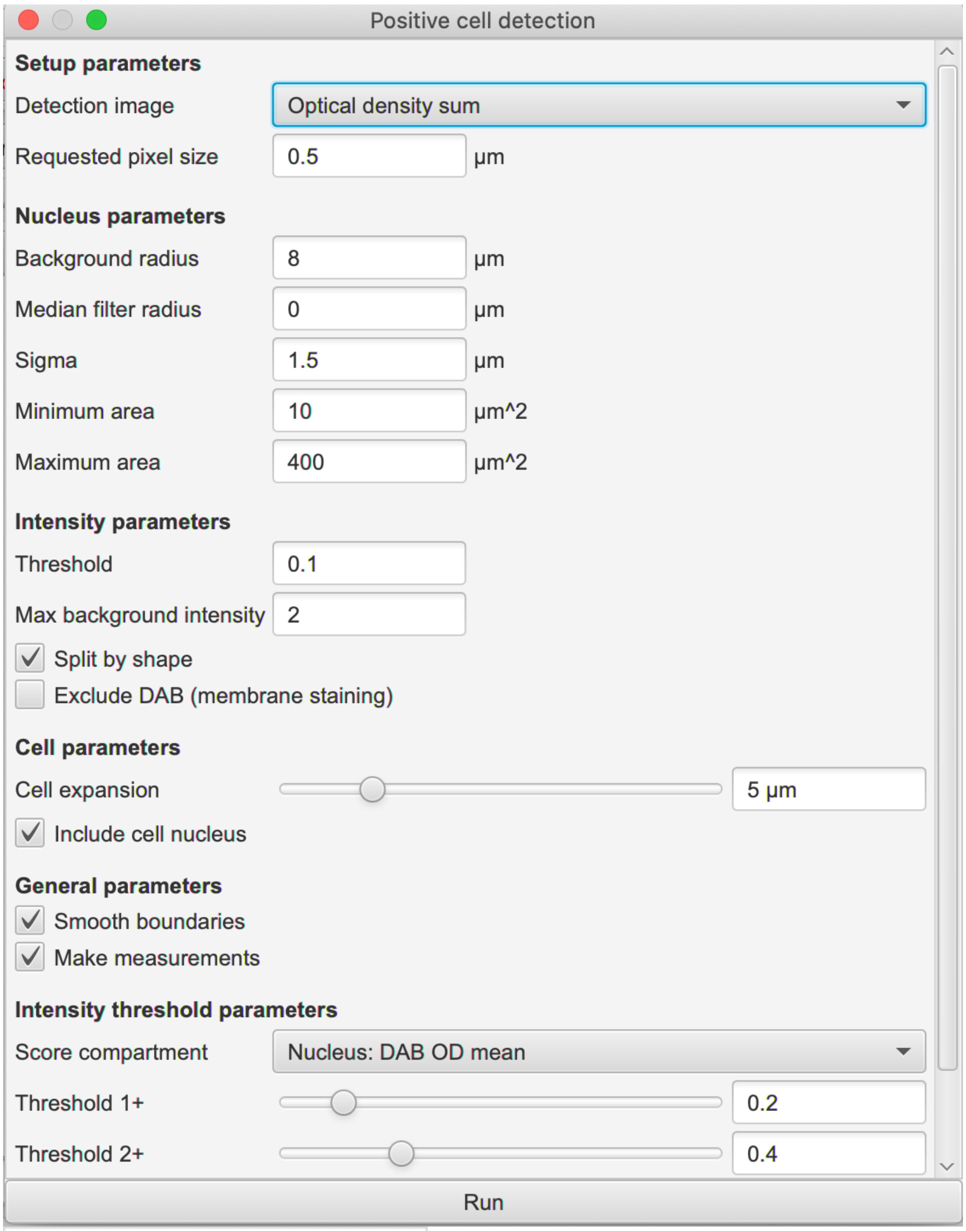

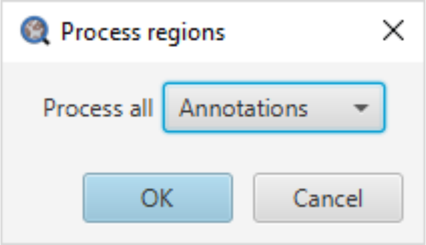

Note: Although default parameters for nuclei detection worked without modification for most cases, for cases with faint Hematoxylin staining or open chromatin pattern (nuclear grade 3), nuclear detection parameters were adjusted empirically to best match the nuclear outline of tumor cells.. It is recommended that one representative tile is selected for cell detection analysis to adjust the parameters before performing analysis of the entire ROI. The result will look like the below captured screen. The ROI will now look blue with yellow grids.

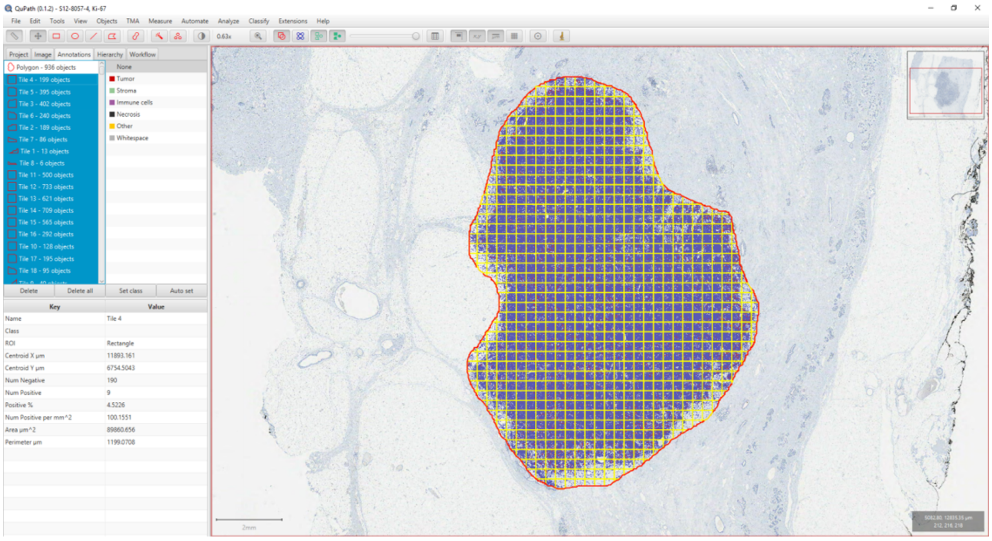

Make sure that the tool bar viewing options are correctly selected to visualize the detection results.

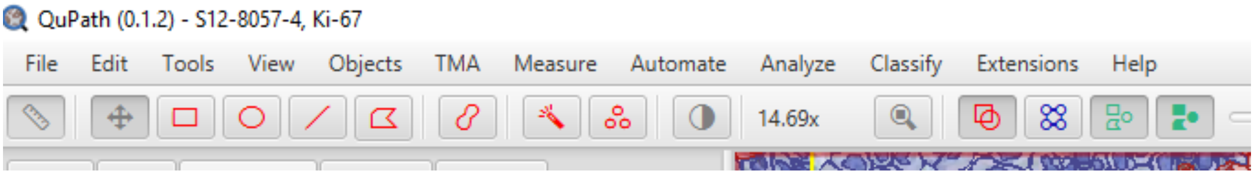

Below is a screen capture of a higher magnification view. Note that both nuclei and cell boundaries are delineated and DAB stained nuclei are in red color.

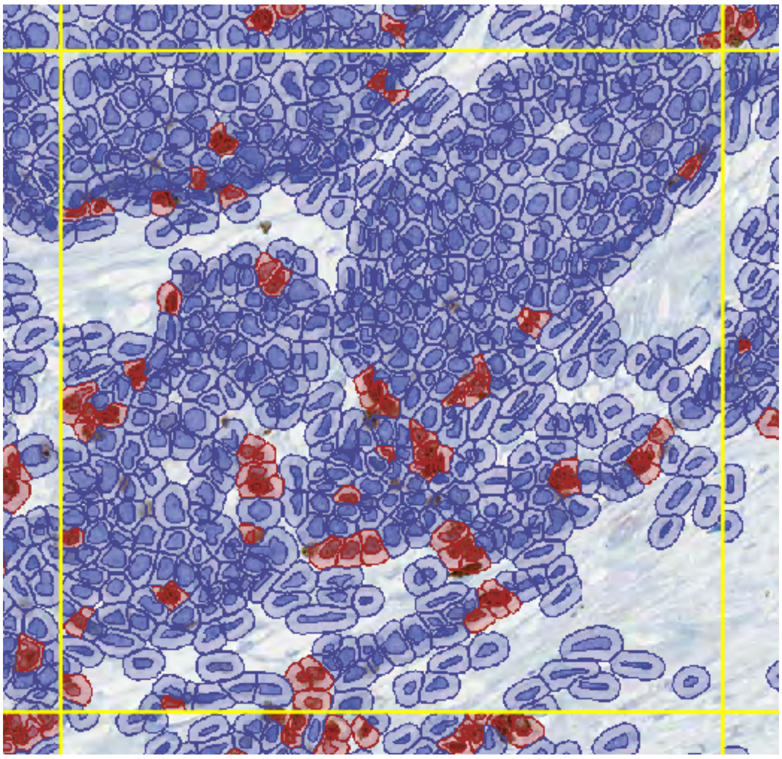

If you want to visualize only nuclei, right click over any detected cell → Cells → Nuclei only
9) if you examine the annotation results at this point, it will look like below; which shows percent positive cells but does not distinguish tumor versus host cells.

Annotation results - S12-8057-4, Ki-67

Name	Class	ROI	Centroid X µm	Centroid Y µm	Num Negative	Num Positive	Positive %	Num Positive per mm^2	Area µm^2	Perimeter µm	Max length µm
Tile 819		Rectangle	15191.9	16950.5	474	29	5.765	322.47	89931.6	1199.5	-
Tile 197		Rectangle	11293.4	10353	704	24	3.297	266.87	89931.6	1199.5	-
Tile 579		Rectangle	10393.7	14551.4	674	33	4.668	366.95	89931.6	1199.5	-
Tile 902		Area (AWT)	10693.9	18449.7	409	17	3.991	189.41	89752.7	1189.3	424.1
Tile 174		Rectangle	10693.6	10053.1	700	21	2.913	233.51	89931.6	1199.5	-
Tile 153		Rectangle	10393.7	9753.2	642	36	5.31	400.3	89931.6	1199.5	-

10) In order to get tumor cell specific labeling index, you have to create a cell classifier and apply the classifier to the entire ROI.
And turn off cell detection view from the tool bar,

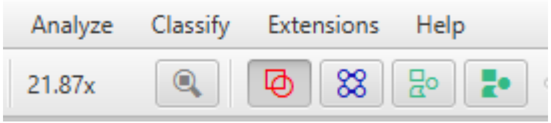

Select any of the selection tools from the tool bar.

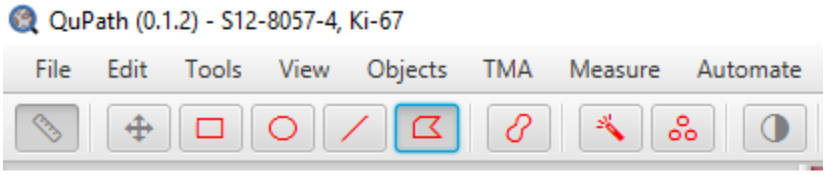

Annotate tumor cells using polygon tool and right click to open pop up menu → set class → tumor. Do the same for stromal cell or other cells that need to be classified differently. Below screen capture shows annotation of both tumor cells (red line) and stromal cells (green line).

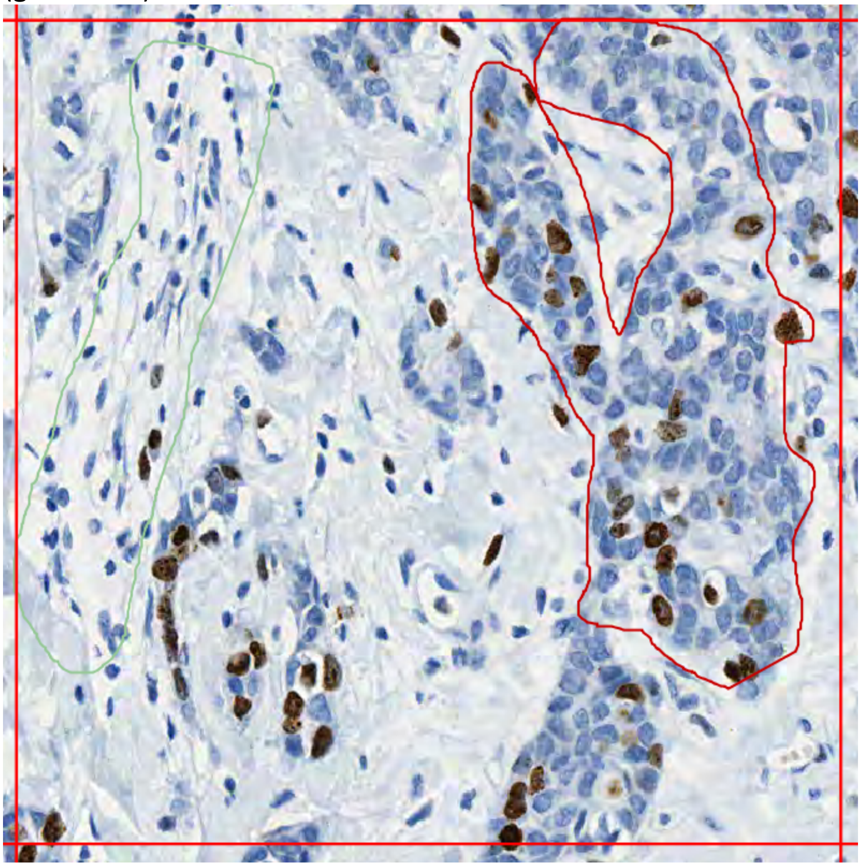

If annotation is performed correctly, the left menu bar annotation tab will show number of tumor cells and stromal cells annotated.

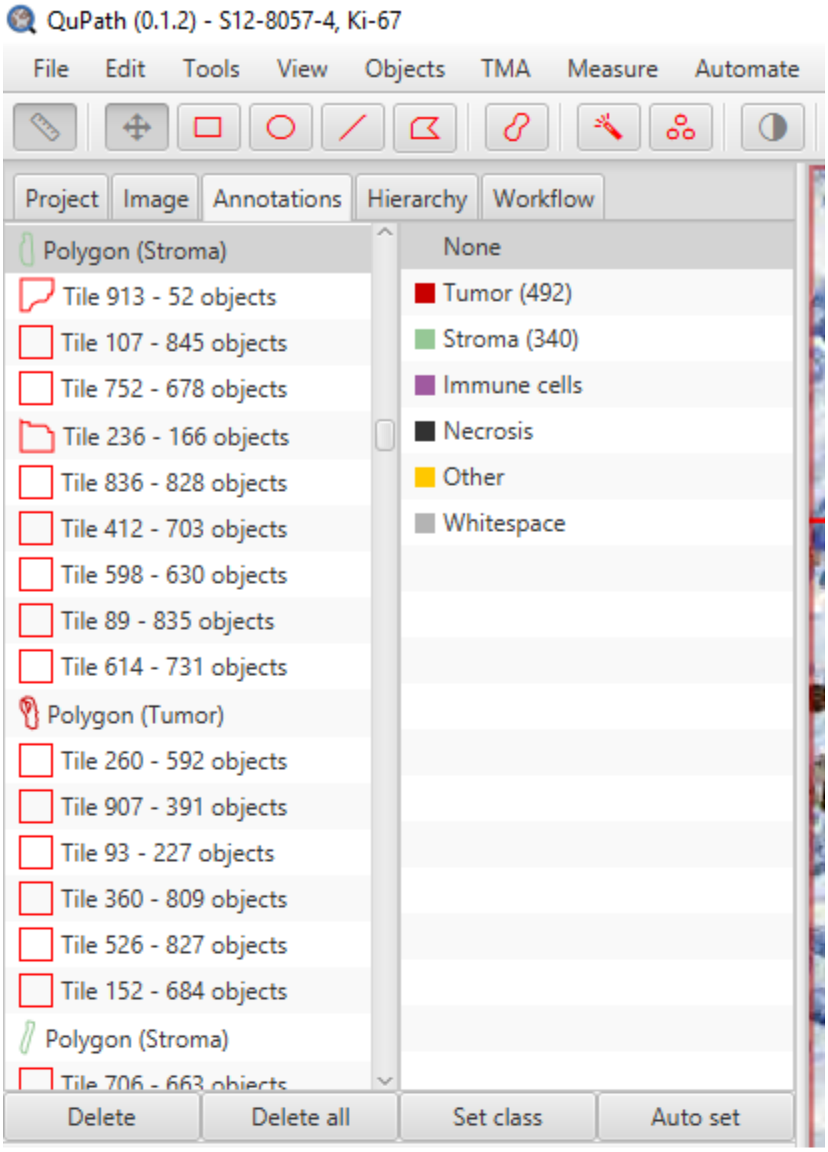

11) Create and apply cell classifier (select Classify from the tool bar → Create detection classifier → select Build & Apply on Create detection classifier pop up window. For intensity features, select Nucleus: DAB OD Mean and check Use Single threshold.

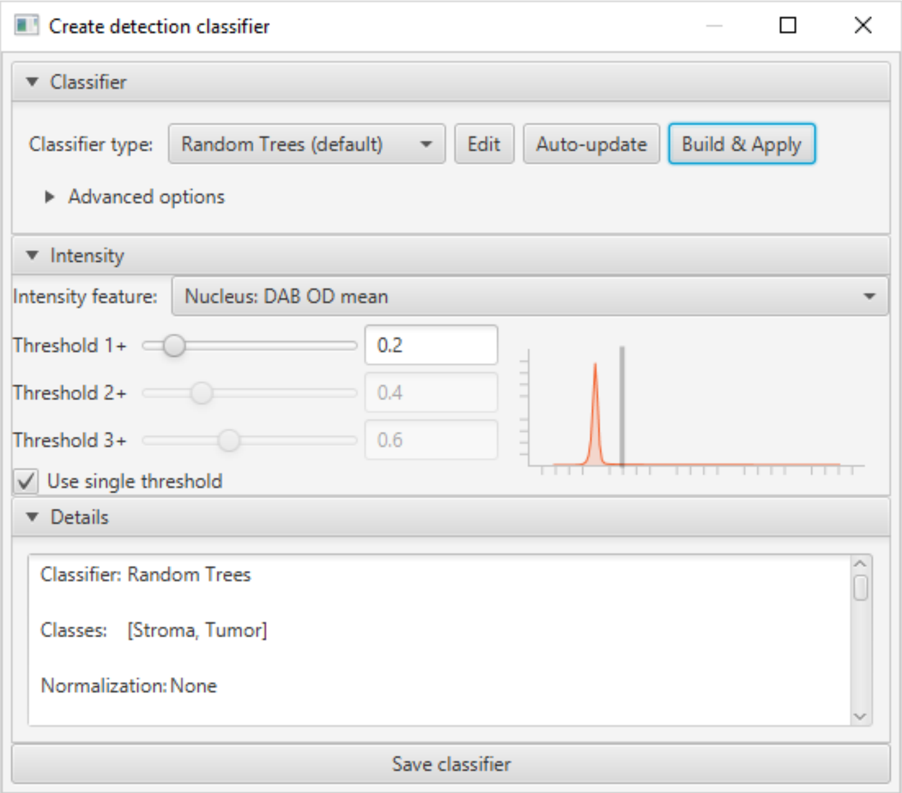
Create detection classifier
Classifier
Classifier type:
Random Trees (default)
Edit
Auto-update
Build & Apply
Advanced options
Intensity
Intensity feature:
Nucleus: DAB OD mean
Threshold 1+
0.2
Threshold 2+
0.4
Threshold 3+
0.6
Use single threshold
Details
Classifier: Random Trees
Classes: [Stroma, Tumor]
Normalization: None
Save classifier

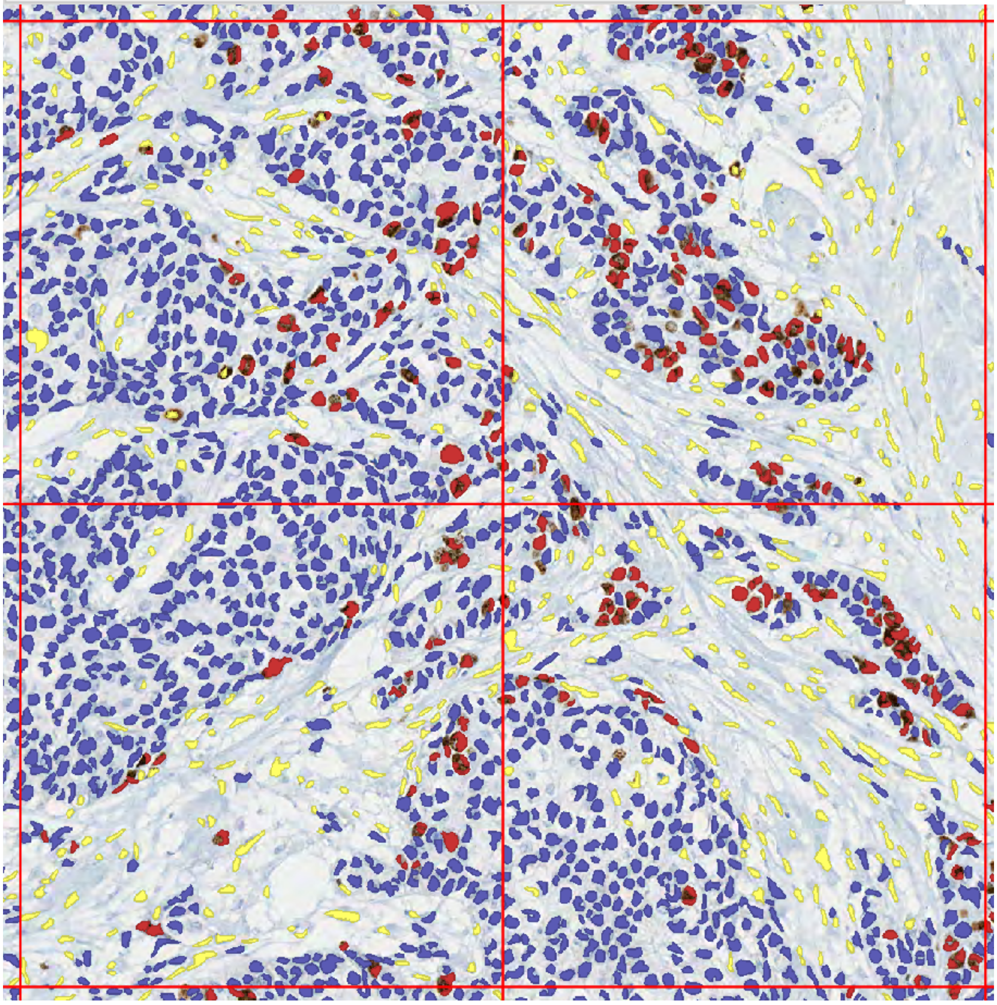

12) Inspect if the cells are correctly classified – if not annotate more cells and reclassify until satisfiable result is obtained. You can save the classifier to apply to other cases – in that case use option load classifier and run.

13) Select the show measurement button from the tool bar → Select show annotation measurements

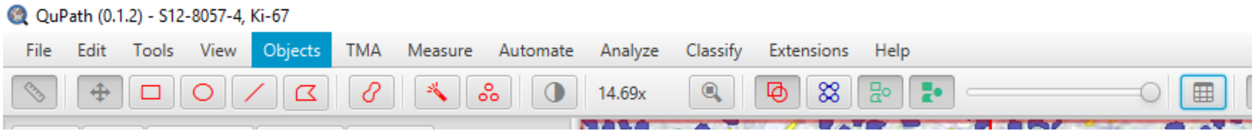

14) Export annotated data to a spreadsheet by clicking on Copy to clipboard and paste to Excel spreadsheet.

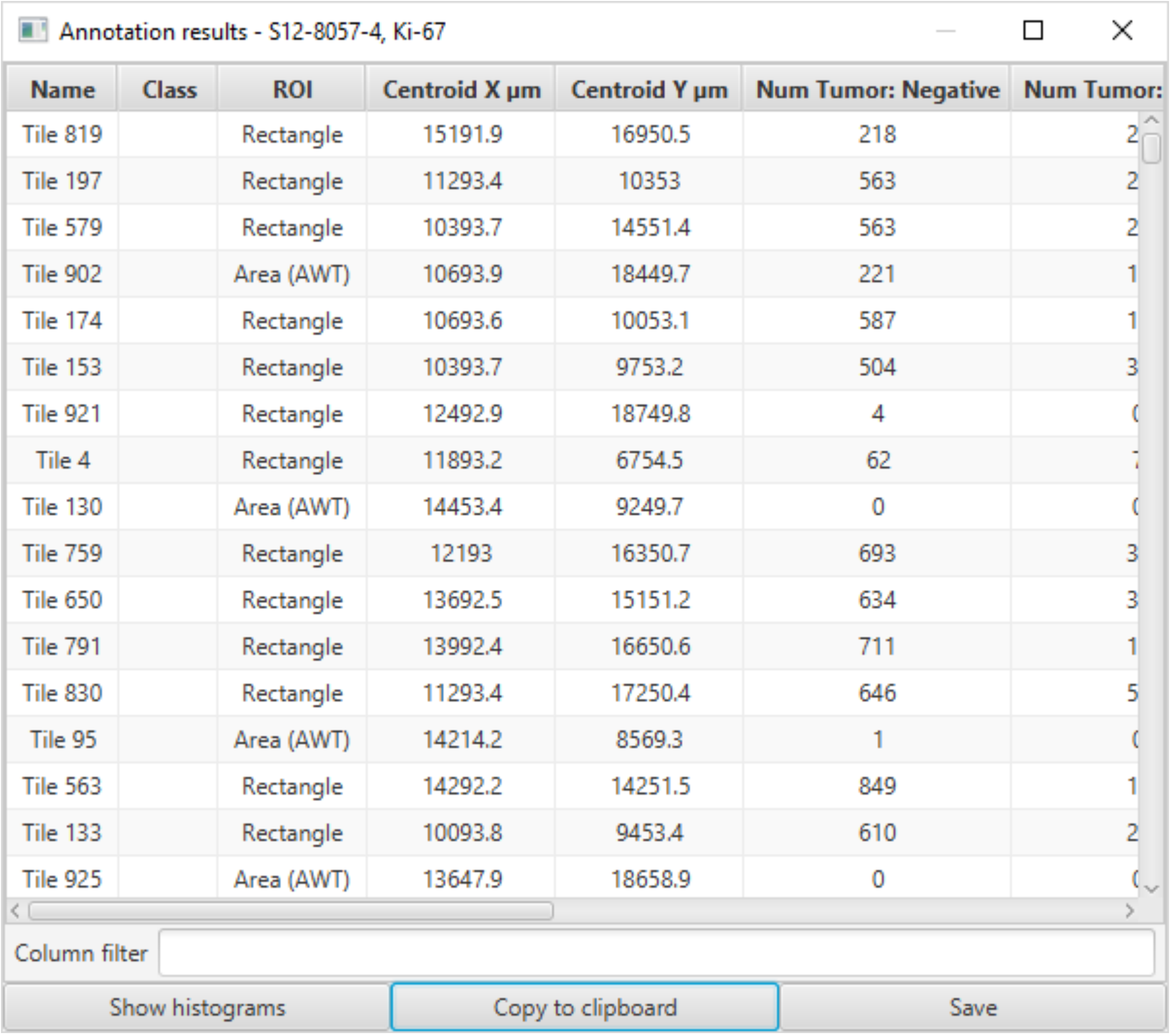
Annotation results - S12-8057-4, Ki-67

Name	Class	ROI	Centroid X µm	Centroid Y µm	Num Tumor: Negative	Num Tumor:
Tile 819		Rectangle	15191.9	16950.5	218	2
Tile 197		Rectangle	11293.4	10353	563	2
Tile 579		Rectangle	10393.7	14551.4	563	2
Tile 902		Area (AWT)	10693.9	18449.7	221	1
Tile 174		Rectangle	10693.6	10053.1	587	1
Tile 153		Rectangle	10393.7	9753.2	504	3
Tile 921		Rectangle	12492.9	18749.8	4	(
Tile 4		Rectangle	11893.2	6754.5	62	7
Tile 130		Area (AWT)	14453.4	9249.7	0	(
Tile 759		Rectangle	12193	16350.7	693	3
Tile 650		Rectangle	13692.5	15151.2	634	3
Tile 791		Rectangle	13992.4	16650.6	711	1
Tile 830		Rectangle	11293.4	17250.4	646	5
Tile 95		Area (AWT)	14214.2	8569.3	1	(
Tile 563		Rectangle	14292.2	14251.5	849	1
Tile 133		Rectangle	10093.8	9453.4	610	2
Tile 925		Area (AWT)	13647.9	18658.9	0	(

Column filter

Show histograms | Copy to clipboard | Save

15) Exclude of grids with less than 100 tumor cells

16) Calculate average score (the highest score is the hottest spot score)

Supplementary Note 2 - R code for data analysis

```
# 300 micron size field processing


data.ki67.raw.300 <- subset(ki67.raw.300,
ki67.raw.300$ROI != "Polygon" & ki67.raw.300$Name !=
"PathAnnotationObject" & ki67.raw.300$`Num Tumor
(base)`>100, na.rm=TRUE)
data.ki67.raw.300$`Tumor: Positive %` <-
as.numeric(data.ki67.raw.300$`Tumor: Positive %` )
ki67.300 <- sort(data.ki67.raw.300$`Tumor: Positive %`)

## average, hottestspot, hotspot(average of top 5)
#All.ki67.300.average <- mean(ki67.300)
All.ki67.300.average <- 100*sum(data.ki67.raw.300$`Num
Tumor: Positive`)/sum(data.ki67.raw.300$`Num Tumor
(base)`)

All.ki67.300.hottestspot <- tail(ki67.300, n=1)
All.ki67.300.hotspot <- mean(tail(ki67.300, n=5))


ki67.300.range <- max(ki67.300) - min(ki67.300)

ki67.300.zero <- 0
ki67.300.low <- ki67.300.range/3
ki67.300.med <- 2*(ki67.300.range/3)

ki67.300.zero.count <- as.data.frame(subset(ki67.300,
ki67.300 == 0))
ki67.300.low.count <- as.data.frame(subset(ki67.300,
ki67.300 <ki67.300.low & ki67.300>0))
ki67.300.med.count <- as.data.frame(subset(ki67.300,
ki67.300 >=ki67.300.low & ki67.300 <ki67.300.med))
ki67.300.high.count <- as.data.frame(subset(ki67.300,
ki67.300 >=ki67.300.med))

ki67.300.zero.percent <- 100*nrow(ki67.300.zero.count)/
nrow(data.ki67.raw.300)
ki67.300.low.percent <- 100*nrow(ki67.300.low.count)/
nrow(data.ki67.raw.300)
ki67.300.med.percent <- 100*nrow(ki67.300.med.count)/
nrow(data.ki67.raw.300)
ki67.300.high.percent <- 100*nrow(ki67.300.high.count)/
```

```
nrow(data.ki67.raw.300)

##### for global average

ki67.300.global.average.repeated <- replicate(1000, {

  ki67.result.300 <- vector()


  if (ki67.300.zero.percent != 0) {
    if (ki67.300.zero.percent>=25) {
      ki67.result.300 <- append(ki67.result.300, 0)
      ki67.result.300 <- append(ki67.result.300, 0)
    } else {
      ki67.result.300 <- append(ki67.result.300, 0)
    }}




  if (ki67.300.low.percent != 0) {
    if (ki67.300.low.percent>=25) {
      ki67.result.300 <- append(ki67.result.300,
sample(ki67.300.low.count$`subset(ki67.300, ki67.300 <
ki67.300.low & ki67.300 > 0)`, 2, replace=FALSE))
    } else {
      ki67.result.300 <-  append(ki67.result.300,
sample(ki67.300.low.count$`subset(ki67.300, ki67.300 <
ki67.300.low & ki67.300 > 0)`, 1, replace=FALSE))
    }}



  if (ki67.300.med.percent != 0) {
    if (ki67.300.med.percent>=25) {
      ki67.result.300 <- append(ki67.result.300,
sample(ki67.300.med.count$`subset(ki67.300, ki67.300 >=
ki67.300.low & ki67.300 < ki67.300.med)`, 2,
replace=FALSE))
    } else {
      ki67.result.300 <- append(ki67.result.300,
sample(ki67.300.med.count$`subset(ki67.300, ki67.300 >=
ki67.300.low & ki67.300 < ki67.300.med)`, 1,
replace=FALSE))
```

```
    }}


  if (ki67.300.high.percent != 0) {
    if (ki67.300.high.percent>=25) {
      ki67.result.300 <- append(ki67.result.300,
sample(ki67.300.high.count$`subset(ki67.300, ki67.300 >=
ki67.300.med)`, 2, replace=FALSE))
    } else {
      ki67.result.300 <-  append(ki67.result.300,
sample(ki67.300.high.count$`subset(ki67.300, ki67.300 >=
ki67.300.med)`, 1, replace=FALSE))
    }}


  ki67.result.300.average <- mean(ki67.result.300)

})

hist(ki67.300.global.average.repeated)
summary(ki67.300.global.average.repeated)

## weighted average

ki67.300.average.weighted.repeated <- replicate(1000, {

  ki67.result.300.zero <- vector()
  ki67.result.300.low <- vector()
  ki67.result.300.med <- vector()
  ki67.result.300.high <- vector()

  if (ki67.300.zero.percent != 0) {
    if (ki67.300.zero.percent>=25) {
      ki67.result.300.zero <- append(ki67.result.300.zero
0)
      ki67.result.300.zero <- append(ki67.result.300.zero
0)
    } else {
      ki67.result.300.zero <- append(ki67.result.300.zero
0)
    }}
  else {ki67.result.300.zero <-
append(ki67.result.300.zero, 0)}
```

```
  if (ki67.300.low.percent != 0) {
    if (ki67.300.low.percent>=25) {
      ki67.result.300.low <- append(ki67.result.300.low,
sample(ki67.300.low.count$`subset(ki67.300, ki67.300 <
ki67.300.low & ki67.300 > 0)`, 2, replace=FALSE))
    } else {
      ki67.result.300.low <-  append(ki67.result.300.low,
sample(ki67.300.low.count$`subset(ki67.300, ki67.300 <
ki67.300.low & ki67.300 > 0)`, 1, replace=FALSE))
    }}

  else ki67.result.300.low <- append(ki67.result.300.low,
0)


  if (ki67.300.med.percent != 0) {
    if (ki67.300.med.percent>=25) {
      ki67.result.300.med <- append(ki67.result.300.med,
sample(ki67.300.med.count$`subset(ki67.300, ki67.300 >=
ki67.300.low & ki67.300 < ki67.300.med)`, 2,
replace=FALSE))
    } else {
      ki67.result.300.med <- append(ki67.result.300.med,
sample(ki67.300.med.count$`subset(ki67.300, ki67.300 >=
ki67.300.low & ki67.300 < ki67.300.med)`, 1,
replace=FALSE))
    }}
  else ki67.result.300.med <- append(ki67.result.300.med,
0)


  if (ki67.300.high.percent != 0) {
    if (ki67.300.high.percent>=25) {
      ki67.result.300.high <- append(ki67.result.300.high
sample(ki67.300.high.count$`subset(ki67.300, ki67.300 >=
ki67.300.med)`, 2, replace=FALSE))
    } else {
      ki67.result.300.high <-
append(ki67.result.300.high,
sample(ki67.300.high.count$`subset(ki67.300, ki67.300 >=
ki67.300.med)`, 1, replace=FALSE))
```

```
    }}

  else ki67.result.300.high <-
append(ki67.result.300.high, 0)

  ki67.result.300.average.weighted <-
(0*ki67.300.zero.percent +
mean(ki67.result.300.low)*ki67.300.low.percent +
mean(ki67.result.300.med)*ki67.300.med.percent  +
mean(ki67.result.300.high)*ki67.300.high.percent)/100


})

hist(ki67.300.average.weighted.repeated)
summary(ki67.300.average.weighted.repeated)
```

Supplementary figure 1.

a

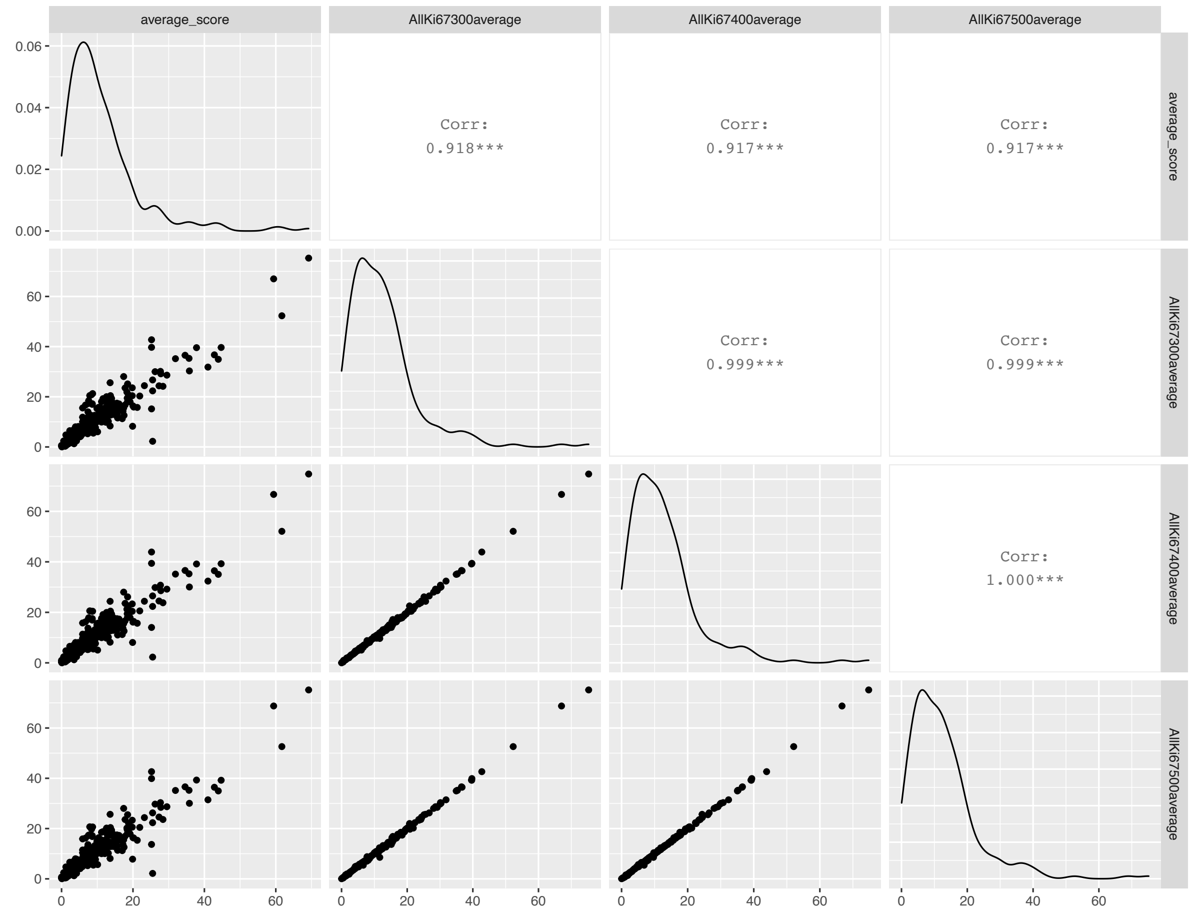

b

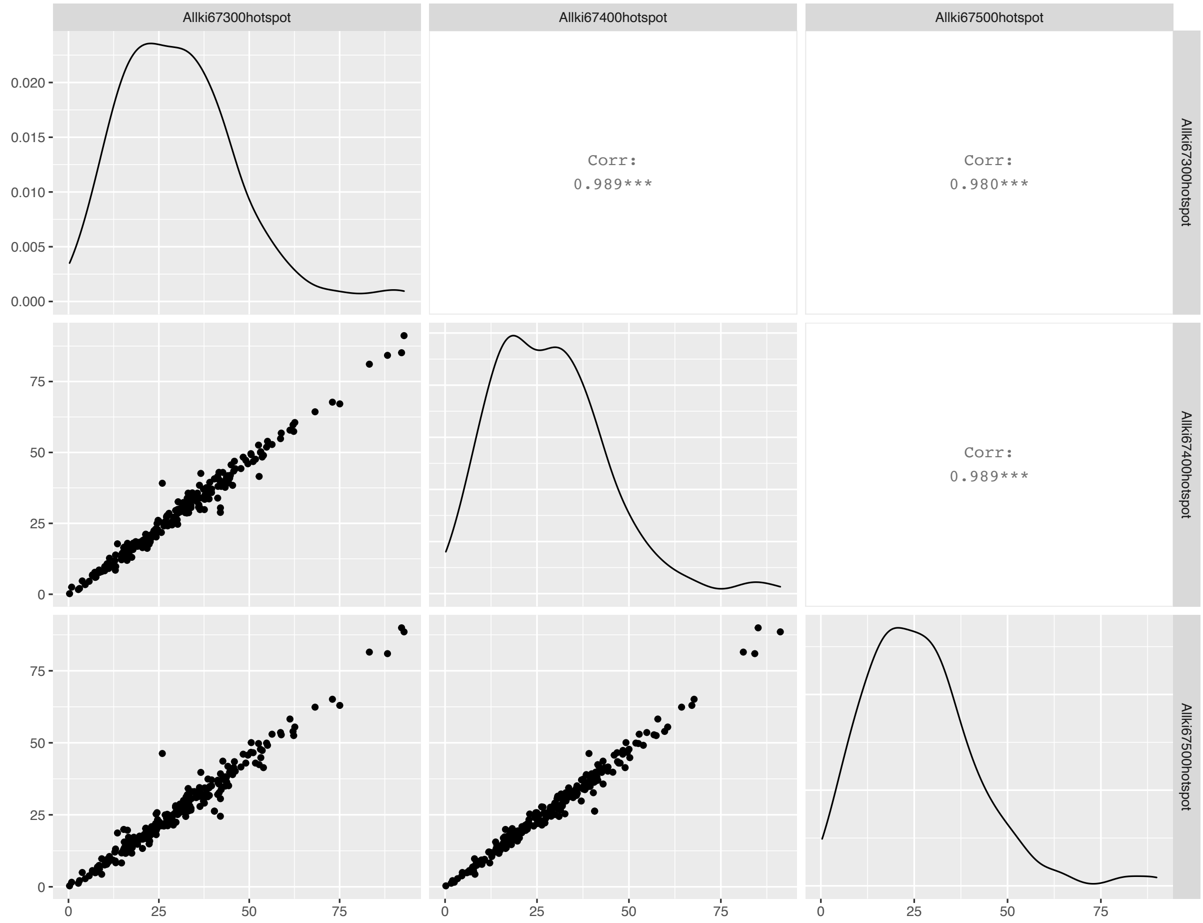

Allki67300hotspot
Allki67400hotspot
Allki67500hotspot
Corr:
0.989***
Corr:
0.980***
Corr:
0.989***
0.020
0.015
0.010
0.005
0.000
75
50
25
0

c

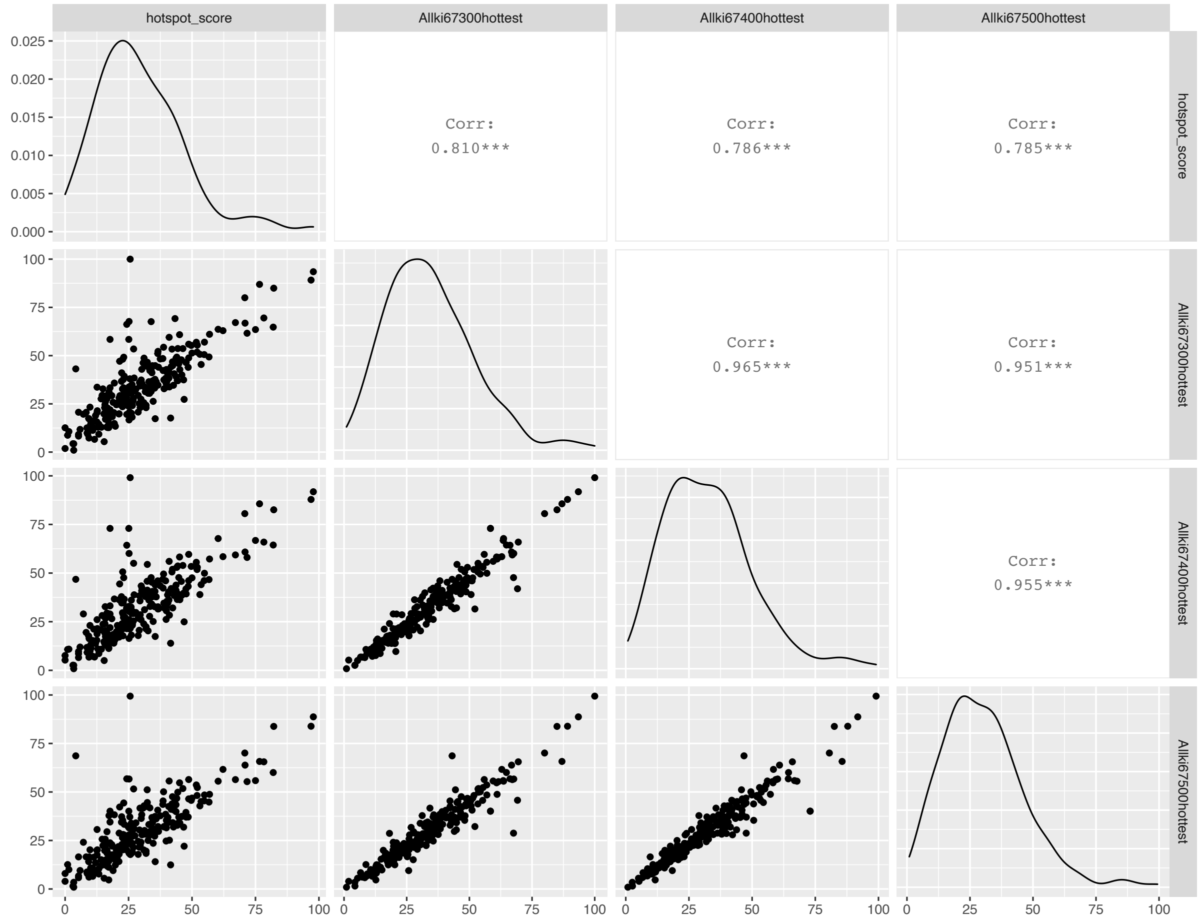

Supplementary figure 1. Reproducibility of image analysis

1a. Correlation among average Ki67 scores from second formal analyses with grid size 300, 400, and 500 micrometers as well as the average score from the first informal analysis (in which grid sizes 300 or 400 were subjectively selected for each case based on tumor cellularity, tumor size, and staining heterogeneity)
Average_score = average score from first informal analysis
ALLKi67300average = average score from the second formal analysis with grid size of 300 micrometers
ALLKi67400average = average score from the second formal analysis with grid size of 400 micrometers
ALLKi67500average = average score from the second formal analysis with grid size of 500 micrometers
Corr = Pearson correlation coefficient

1b. Correlation among hostspot Ki67 scores from analyses with grid size 300, 400, and 500 micrometers.

ALLKi67300hotspot = hotspot score from the second formal analysis with grid size of 300 micrometers
ALLKi67400hotspot = hotspot score from the second formal analysis with grid size of 400 micrometers
ALLKi67500hotspot = hotspot score from the second formal analysis with grid size of 500 micrometers

1c. Correlation among hottest Ki67 scores from second formal analyses with grid size 300, 400, and 500 micrometers as well as the hottest score from the first informal analysis (in which grid sizes 300 or 400 were subjectively selected for each case based on tumor cellularity, tumor size, and staining heterogeneity).

hotspot_score = hottest spot score from first informal analysis
ALLKi67300hottest = hottest spot score from the second formal analysis with grid size of 300 micrometers
ALLKi67400hottest = hottest spot score from the second formal analysis with grid size of 400 micrometers
ALLKi67500hottest = hottest spot score from the second formal analysis with grid size of 500 micrometers

Supplementary figure 2.

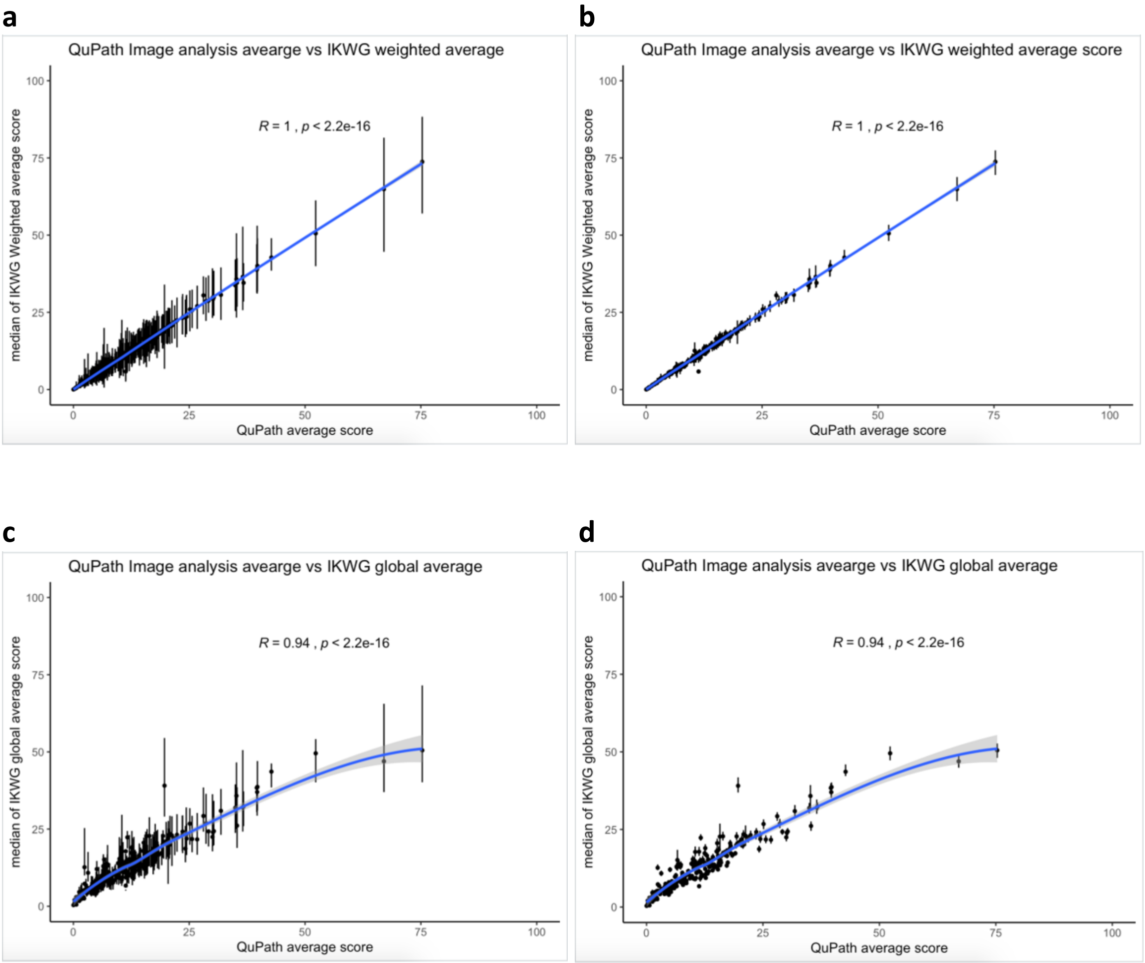

Supplementary figure 2. Simulation of International Ki67 Working Group scoring method

2a. Correlation between IKWG weighted average score and QuPath average score (error bars of the left plot represent minimum-maximum range from 1,000 simulations)

2b. Correlation between IKWG weighted average score and QuPath average score (error bars represent interquartile range from 1,000 simulations)

2c. Correlation between IKWG global average score and QuPath average score (error bars of the left plot represent minimum-maximum range from 1,000 simulations)

2d. Correlation between IKWG global average score and QuPath average score (error bars of the right plot represent interquartile range from 1,000 simulations)

Supplementary figure 3.

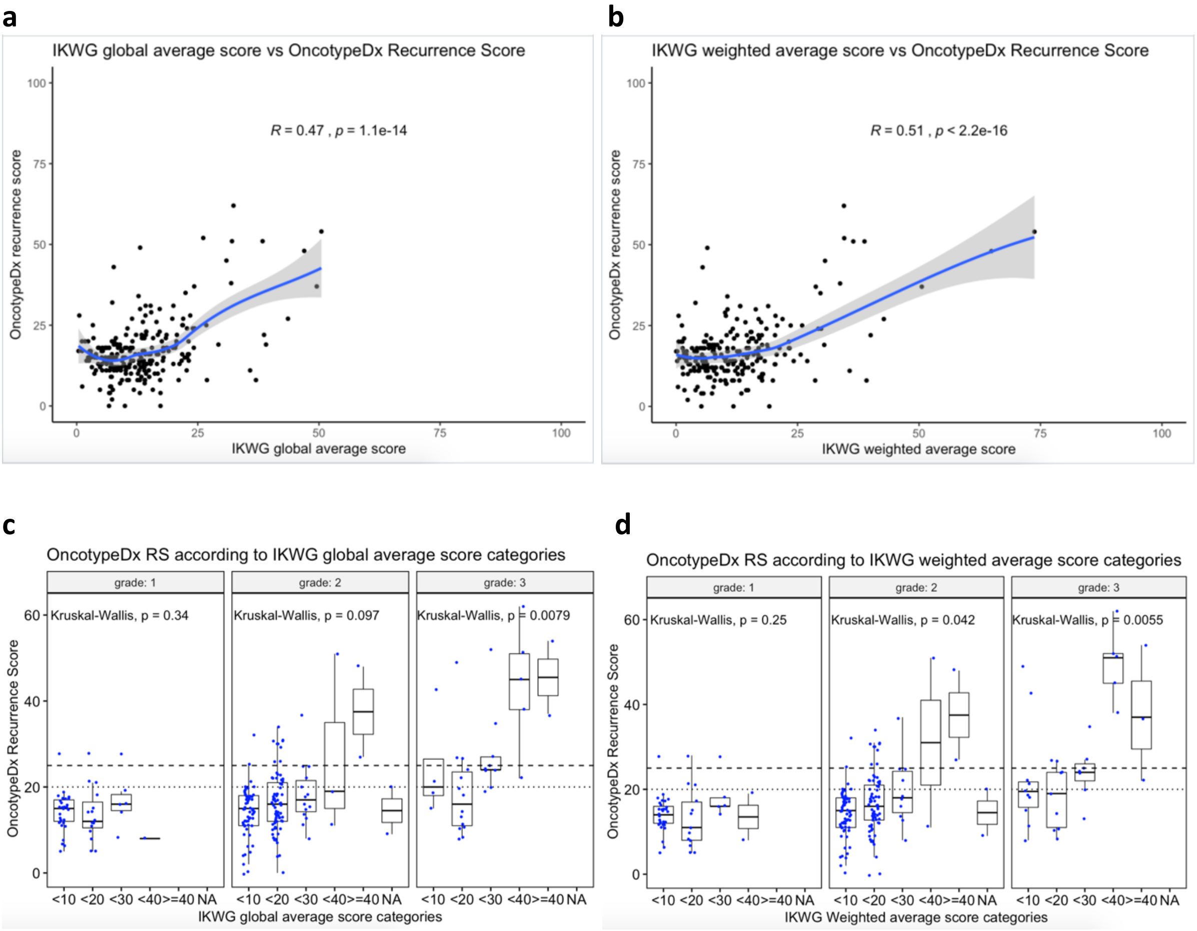

Supplementary figure 3. Correlation between the International Ki67 Working Group average scores and OncotypeDx recurrence score

3a. Correlation between the IKWG global average score (median of 1,000 simulations) as a continuous variable and OncotypeDx recurrence score

3b. Correlation between the IKWG weighted average score (median of 1,000 simulations) as a continuous variable and OncotypeDx recurrence score

3c. Correlation between the IKWG global average score (median of 1,000 simulations) as a categorical variable and OncotypeDx recurrence score within each tumor histological grade category (broken horizontal line: OncotypeDx RS =25) (note: NA represents two cases for which simulation could not be performed due to small tumor area). Box plot is drawn as follows; each box represents interquartile range with the inside line representing median value. Whiskers represent maximum (75th percentile + 1.5*interquartile range) and minimum (25th percentile – 1.5*interquartile range) values. Each dot represents individual samples.

3d. Correlation between the IKWG weighted average score (median of 1,000 simulations) as a categorical variable and OncotypeDx recurrence score within each tumor histological grade category (broken horizontal line: OncotypeDx RS =25) (note: NA represents two cases for which simulation could not be performed due to small tumor area).

Supplementary figure 4.

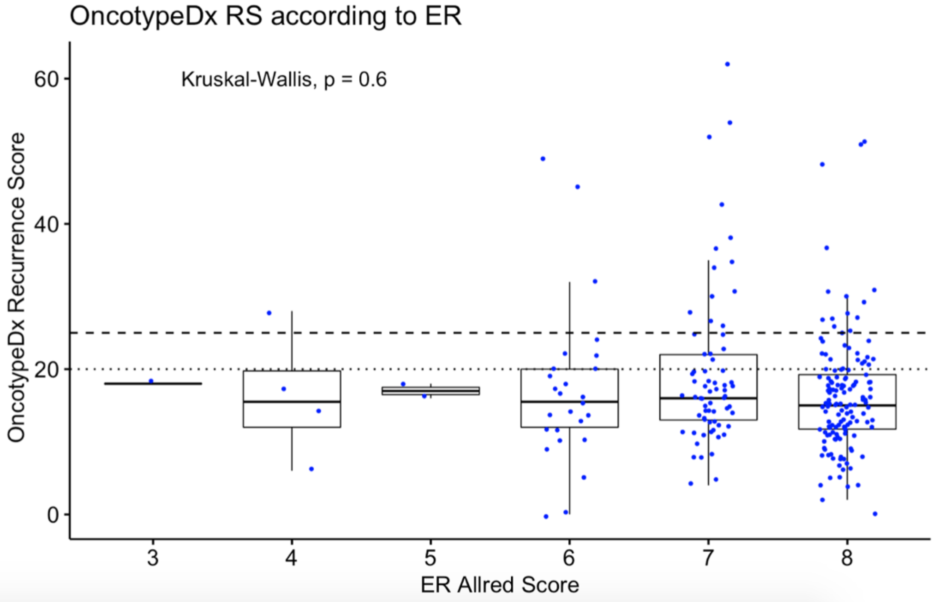

Supplementary figure 4. Correlation between estrogen receptor levels and OncotypeDx RS. Box plot is drawn as follows; each box represents interquartile range with the inside line representing median value. Whiskers represent maximum (75th percentile + 1.5*interquartile range) and minimum (25th percentile – 1.5*interquartile range) values. Each dot represents individual samples. Broken horizontal line represents OncotypeDx RS 25.

Supplementary figure 5.

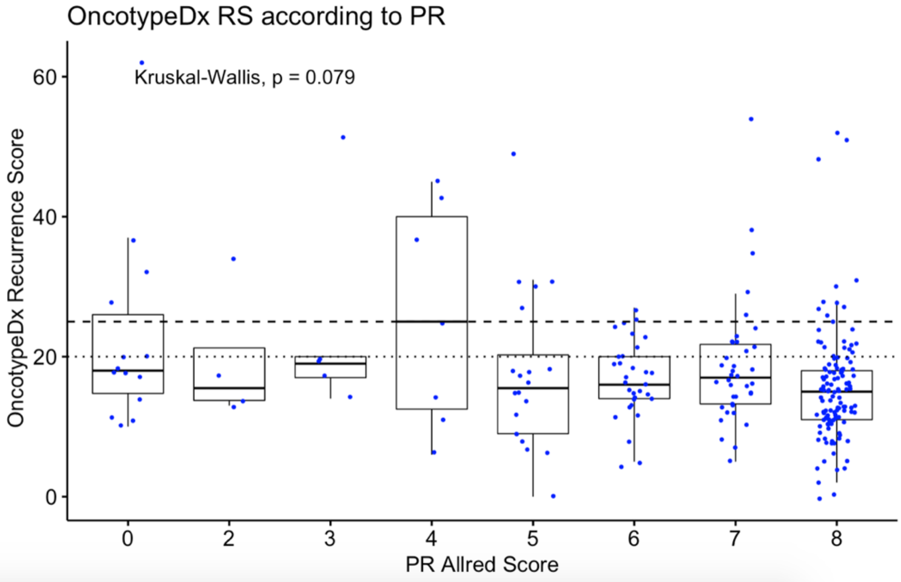

Supplementary figure 4. Correlation between progesterone receptor levels and OncotypeDx RS. Box plot is drawn as follows; each box represents interquartile range with the inside line representing median value. Whiskers represent maximum (75th percentile + 1.5*interquartile range) and minimum (25th percentile – 1.5*interquartile range) values. Each dot represents individual samples. Broken horizontal line represents OncotypeDx RS 25.
