# Supplementary material for: Systematic evaluation of scoring methods for Ki67 as a surrogate for 21-gene recurrence score
Source: NPJ Breast Cancer. 2021 Feb 12;7:13. doi: 10.1038/s41523-021-00221-z (PMC7881194; doi:10.1038/s41523-021-00221-z)
Supplement: Supplementary file 2 — Reporting Summary Checklist [file 41523_2021_221_MOESM2_ESM.pdf]

nature research

Corresponding author(s): Soonmyung Paik, Eun Sook Lee

Last updated by author(s): Dec 14, 2020

# Reporting Summary

Nature Research wishes to improve the reproducibility of the work that we publish. This form provides structure for consistency and transparency in reporting. For further information on Nature Research policies, see our Editorial Policies and the Editorial Policy Checklist.

## Statistics

For all statistical analyses, confirm that the following items are present in the figure legend, table legend, main text, or Methods section.

| n/a | Confirmed | |
|---|---|---|
| ☐ | ☒ | The exact sample size (*n*) for each experimental group/condition, given as a discrete number and unit of measurement |
| ☐ | ☒ | A statement on whether measurements were taken from distinct samples or whether the same sample was measured repeatedly |
| ☐ | ☒ | The statistical test(s) used AND whether they are one- or two-sided<br>*Only common tests should be described solely by name; describe more complex techniques in the Methods section.* |
| ☐ | ☒ | A description of all covariates tested |
| ☐ | ☒ | A description of any assumptions or corrections, such as tests of normality and adjustment for multiple comparisons |
| ☐ | ☒ | A full description of the statistical parameters including central tendency (e.g. means) or other basic estimates (e.g. regression coefficient) AND variation (e.g. standard deviation) or associated estimates of uncertainty (e.g. confidence intervals) |
| ☐ | ☒ | For null hypothesis testing, the test statistic (e.g. *F*, *t*, *r*) with confidence intervals, effect sizes, degrees of freedom and *P* value noted<br>*Give P values as exact values whenever suitable.* |
| ☒ | ☐ | For Bayesian analysis, information on the choice of priors and Markov chain Monte Carlo settings |
| ☒ | ☐ | For hierarchical and complex designs, identification of the appropriate level for tests and full reporting of outcomes |
| ☐ | ☒ | Estimates of effect sizes (e.g. Cohen's *d*, Pearson's *r*), indicating how they were calculated |

*Our web collection on statistics for biologists contains articles on many of the points above.*

## Software and code

Policy information about availability of computer code

| | |
|---|---|
| Data collection | QuPath version 0.2.1<br>R version 3.6.3 |
| Data analysis | R version 3.6.3 |

For manuscripts utilizing custom algorithms or software that are central to the research but not yet described in published literature, software must be made available to editors and reviewers. We strongly encourage code deposition in a community repository (e.g. GitHub). See the Nature Research guidelines for submitting code & software for further information.

## Data

Policy information about availability of data

All manuscripts must include a data availability statement. This statement should provide the following information, where applicable:

- Accession codes, unique identifiers, or web links for publicly available datasets
- A list of figures that have associated raw data
- A description of any restrictions on data availability

The scanned whole slide image files and individual QuPath image analysis result files are available upon reasonable request.

# Field-specific reporting

Please select the one below that is the best fit for your research. If you are not sure, read the appropriate sections before making your selection.

☒ Life sciences ☐ Behavioural & social sciences ☐ Ecological, evolutionary & environmental sciences

For a reference copy of the document with all sections, see nature.com/documents/nr-reporting-summary-flat.pdf

# Life sciences study design

All studies must disclose on these points even when the disclosure is negative.

| | |
|---|---|
| Sample size | All available cases with associated data were included |
| Data exclusions | none |
| Replication | All cases were analyzed twice with QuPath image analysis, with one year between the two analyses. |
| Randomization | none |
| Blinding | The pathologist performing QuPath image analysis was blinded to OncotypeDx results. |

# Reporting for specific materials, systems and methods

We require information from authors about some types of materials, experimental systems and methods used in many studies. Here, indicate whether each material, system or method listed is relevant to your study. If you are not sure if a list item applies to your research, read the appropriate section before selecting a response.

## Materials & experimental systems

| n/a | Involved in the study | |
|---|---|---|
| ☐ | ☒ | Antibodies |
| ☒ | ☐ | Eukaryotic cell lines |
| ☒ | ☐ | Palaeontology and archaeology |
| ☒ | ☐ | Animals and other organisms |
| ☐ | ☒ | Human research participants |
| ☐ | ☒ | Clinical data |
| ☒ | ☐ | Dual use research of concern |

## Methods

| n/a | Involved in the study | |
|---|---|---|
| ☒ | ☐ | ChIP-seq |
| ☒ | ☐ | Flow cytometry |
| ☒ | ☐ | MRI-based neuroimaging |

## Antibodies

| | |
|---|---|
| Antibodies used | ER (SP1, Ventana, RTU), PR(1E2, Ventana, RTU), Ki-67 (MIB-1, DAKO, 1:200 dilution) |
| Validation | anti-ER and anti-PR antibodies are FDA approved IVD reagents provided by Ventana in a Ready to Use reagent.<br>anti-Ki67 antibody clone MIB-1 from DAKO is a widely used antibody and its specificity was validated by exclusive nuclear staining in tumor cells, lymphoid follicles, and no staining in resting normal cells. |

## Human research participants

Policy information about studies involving human research participants

| | |
|---|---|
| Population characteristics | A total of 240 subjects with ER positive breast cancer were analyzed in the study. All patients diagnosed of estrogen receptor positive breast cancer at Korean National Cancer Center and tested for OncotypeDx between 2011 and 2015 were included in the study. |
| Recruitment | All patients diagnosed of estrogen receptor positive breast cancer at Korean National Cancer Center and tested for OncotypeDx between 2011 and 2015 were included in the study. |
| Ethics oversight | This study was approved by the institutional review board of Yonsei University College of Medicine (1-2014-0067). |

Note that full information on the approval of the study protocol must also be provided in the manuscript.

# Clinical data

Policy information about clinical studies

All manuscripts should comply with the ICMJE guidelines for publication of clinical research and a completed CONSORT checklist must be included with all submissions.

| | |
|---|---|
| Clinical trial registration | NA |
| Study protocol | NA |
| Data collection | NA |
| Outcomes | NA |
